# Supplementary material for: Modelling mass drug administration strategies for reducing scabies burden in Monrovia, Liberia
Source: Epidemiol Infect. 2023 Aug 18;151:e153. doi: 10.1017/S0950268823001310 (PMC10548539; doi:10.1017/S0950268823001310)
Supplement: Tellioglu et al. supplementary material [file S0950268823001310sup001.docx]

**Modelling mass drug administration for reducing scabies burden in Monrovia, Liberia**

**Supplementary material**

Nefel Tellioglu^1^, Rebecca H. Chisholm^2,3^, Patricia Therese Campbell^3,4^, Shelui Collinson^5^, Joseph Timothy^6^, Karsor Kollie^7^, Samuel Zayzay^7^, Angela Devine^3,8^, Jodie McVernon^4,9^, Michael Marks^5,10,11^, Nicholas Geard ^1*^

^1^ School of Computing and Information Systems, The University of Melbourne, Melbourne, Australia

^2^ Department of Mathematical and Physical Sciences, La Trobe University, Bundoora, Australia

^3^ Centre for Epidemiology and Biostatistics, Melbourne School of Population and Global Health, The University of Melbourne, Melbourne, Australia

^4^ Department of Infectious Diseases, University of Melbourne, at the Peter Doherty Institute for Infection and Immunity, Melbourne, Victoria, 3000, Australia

^5^ Clinical Research Department, Faculty of Infectious and Tropical Diseases, London School of Hygiene & Tropical Medicine, London, UK

^6^ Department of Disease Control, Faculty of Infectious and Tropical Diseases, London School of Hygiene & Tropical Medicine, London, UK

^7^ Ministry of Health, Monrovia, Liberia

^8^ Global and Tropical Health Division, Menzies School of Health Research, Charles Darwin University, Darwin, Australia

^9^ Victorian Infectious Diseases Reference Laboratory, The Royal Melbourne Hospital, at the Peter Doherty Institute for Infection and Immunity, Melbourne, Australia

^10^ Hospital for Tropical Diseases, University College London Hospital, London, UK

^11^ Division of Infection and Immunity, University College London, London, UK

^*^ Corresponding Author: [ngeard@unimelb.edu.au](mailto:ngeard@unimelb.edu.au)

Table of Contents

[S1. Agent-based model 3](#_Toc136018702)

[S2. Calibration of the agent-based model 4](#_Toc136018703)

[S2.1. Calibration of the population model 4](#_Toc136018704)

[S2.2. Calibration of the disease model 6](#_Toc136018705)

[S3. R_0_ Estimation 10](#_Toc136018706)

[S4. Estimation of disability-adjusted life-years (DALYs) 11](#_Toc136018707)

[S5. Sensitivity analysis of duration of infestation 11](#_Toc136018708)

[S6. Additional MDA results 17](#_Toc136018709)

[S6.1. Results with various treatment efficacies 20](#_Toc136018710)

[**S6.1.1. Results with treatment efficacy of 85% (rather than 90%)** 20](#_Toc136018711)

[**S6.1.2. Results with treatment efficacy of 95% (rather than 90%)** 22](#_Toc136018712)

[**S6.1.3. Results with individuals not receiving one or two doses of an MDA through lower treatment efficacy** 25](#_Toc136018713)

[S6.2. Results with scabies importation 26](#_Toc136018714)

[S6.2.1. Results with one scabies case importation each week 26](#_Toc136018715)

[S6.2.2. Results with five scabies case importation each week 28](#_Toc136018716)

[S7. Analysis of non-compliance and non-treatment 29](#_Toc136018717)

[S7.1 Analysis of systematic non-treatment 29](#_Toc136018718)

[S7.2 Analysis of systematic non-compliance 30](#_Toc136018719)

# **S1. Agent-based model**

We use the disease transmission model with age and household structure from Geard, et al

[1]. In this model, a synthetic population is generated with given demographic parameters. Then, disease transmission is simulated in the generated synthetic population. Using this framework, disease transmission can be simulated over an extended period of time (e.g., decades) with plausible age and household dynamics.

We modelled scabies transmission using an SIS model, where infectious individuals return to the susceptible class on recovery [2]. The infectious duration is sampled from an Erlang distribution (with shape parameter k = 3) and a given mean infectious duration. Two types of transmission are simulated in the model: household and community transmission. In addition, there is an external exposure rate that simulates people becoming infected from a source outside of the model population. The model assumes that a person makes contact with all other members of their household in every time step and that they make contact with other people in the community at age-specific rates. These age-specific community contact rates are represented as a contact matrix which is calculated by the activity levels of age groups. The activity levels are given as the difference between the "all contacts" matrix and the "household contacts" matrix for Liberia by Prem, et al [3] since household contacts are represented explicitly in the model.


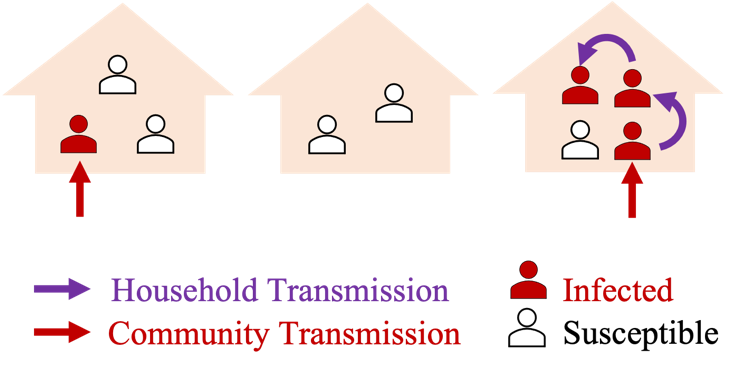


**Fig. S1:** Illustration of community and household transmission in our model. The source (infector) in a community transmission is unknown but the source in a household transmission is known.

The probability of a person becoming infected in each time step is given by:

$$Probability of infection=1 - e^{-{foi}_{iNH}}$$

where *foi* is the force of infection on an individual in age class *i*, belonging to a household of

size 𝑁_H_ [1] The force of infection *foi* is made up of components related to household and community transmission:

$${foi}_{iNH}= q_{h} * \frac{I_{H}}{\left( N_{H} -1 \right)} + q_{C}*\sum_{j=1} \eta_{ij}\frac{I_{j}}{N_{j}}$$

Household Transmission

Community Transmission

where:

- $q_{h}$ = Household transmission coefficient
- $q_{C}$ = Community transmission coefficient
- $\eta_{ij}$ = Number of daily community contacts between age groups *i* and *j*
- $I_{H}$ = Number of infected individuals in a household
- $I_{j}$ = Number of infected individuals in age group *j*

In addition to the endemic transmission, there is also a chance of susceptible individuals being infected by external sources with a probability of external exposure rate multiplied by population size. The rate of household transmission depends on the proportion of infectious people in the household. The rate of community transmission depends on the age-dependent community contact rates 𝜂_ij_ and the proportion of infected people in each age class.

# **S2. Calibration of the agent-based model**

A cross-sectional survey conducted in Monrovia, Liberia in 2020 found a community-level scabies prevalence of 9.3% [4]. While the survey provides a detailed picture of scabies prevalence in sub-Saharan Africa, it is cross-sectional and hence provides only limited information about transmission dynamics and the likely efficacy of control strategies. Since the prevalence is in the range of 2–10%, it is not clear whether an MDA should be applied in Monrovia, and whether an MDA strategy would be effective to reduce the scabies burden.

We first calibrated the population model to the demographic characteristics of the peri-urban settings of Liberia (Section S2.1). Then, we calibrated the disease dynamics to the survey data (Section S2.2).

## **S2.1. Calibration of the population model**

We first calibrated the population demography in our model to match that of the study population [5] and compared characteristics of age and household observed in the synthetic population with the survey data from Monrovia [4] (**Table S*1***). We run the population model for 100 years starting from an initial population size of 5000. The population size became ~13600 after the 100-year burn-in period.

However, the Liberia population characteristics were different from the survey data (**Fig. S*2***). Therefore, we decided to use the death rates [6] and fertility rates [7] of Zambia as these datasets provided a better representation of household- and age-specific characteristics of the survey data collected in New Kru Town, Monrovia [4]. The age and household-size distributions of the synthetic population and original dataset are shown in **Fig. S3** and **Fig. S*4***. The synthetic population in our model represents the demographic characteristics of the survey data.


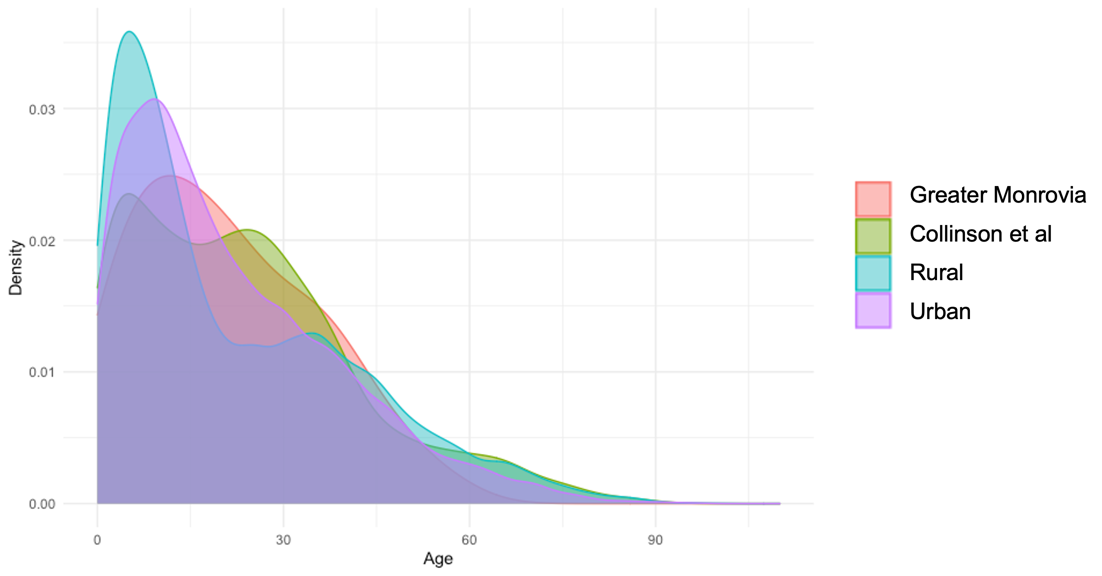


**Fig. S2:** Comparison of age distribution in survey data collected in New Kru Town, Monrovia (Collinson et al - green) [4] and age distributions for Greater Monrovia (red), rural (blue), urban (purple) taken from the World Bank data [5].

**Table S1:** Population model parameters

| **Parameter** | **Description** | **Values** |
| --- | --- | --- |
| Initial population size | Number of individuals in population at start of simulation. | 5000 |
| Population growth rate | Annual rate of change in population size due to natural increase. | 0.01 |
| Immigration rate | Annual rate of change in population size due to immigration. | 0.0 |
| Age- and sex-specific mortality probabilities | Annual probabilities of death by sex and year of age. | Dataset |
| Age-specific relative fertility probabilities | Relative probabilities, given the birth of a child, that the mother is of a specified age. | Dataset |
| Birth gap (mean and SD) | Parameters governing the minimum inter-birth interval. | (270, 0) |
| Couple formation parameters | Age range that a currently single individual is eligible to form a couple, and annual probability that this will occur. | (15, 60), 0.039 |
| Partner age difference (mean and SD) | Parameters governing the sex-dependent age difference between partners during couple formation. | (2, 2) |
| Couple dissolution parameters | Age range that a currently coupled individual is eligible to dissolve that couple, and annual probability that this will occur. | (18, 60), 0.001 |
| Leaving home parameters | Minimum age at which an individual currently living with a parent/guardian will form a new single-person household, and annual probability that this will occur. | 18, 0.008 |


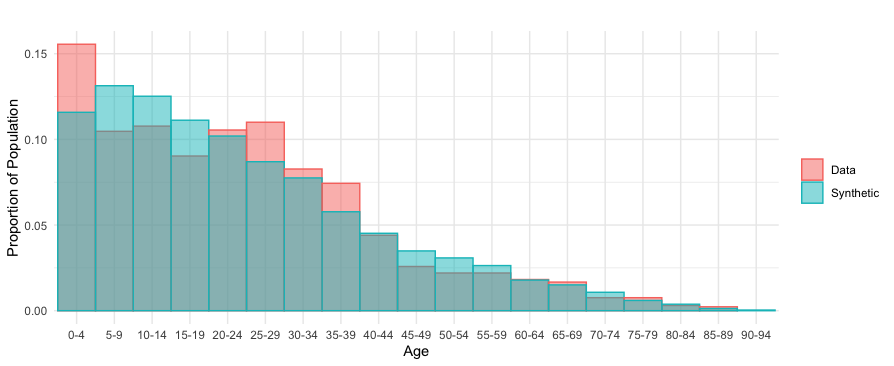


**Fig. S3:** Age Distribution of the Synthetic Population (blue) and Monrovia Dataset (red) at year 0 after 100-year burn-in period


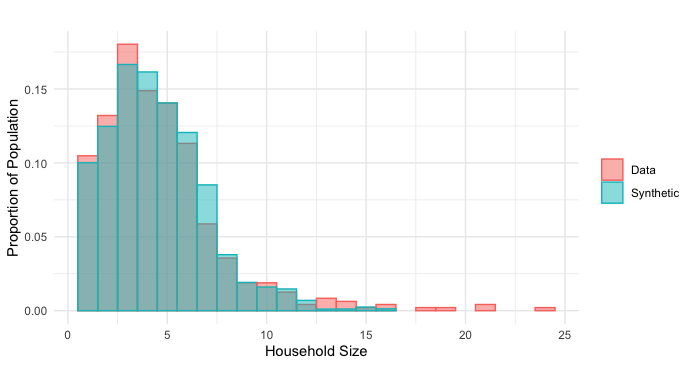


**Fig. S4:** Household Size Distribution of the Synthetic Population (blue) and Monrovia Dataset (red) at year 0 after 100-year burn-in period

## **S2.2. Calibration of the disease model**

This section summarises the calibration of our age and household structured model to data from the household scabies survey conducted in Monrovia, Liberia. We calibrated household and community transmission coefficients to match the age and household size specific patterns of scabies incidence. We applied The Bayesian Optimization in Likelihood-Free Inference (BOLFI) framework to calibrate the scabies model to the Liberian population [8]. To begin, we assumed a mean duration of infestation of 90 days which represents the time until seeking treatment. We used BOLFI to calibrate two unknown parameters: household and community transmission coefficients (*q_h_* and *q*). Using BOLFI involves defining a set of summary statistics that can be calculated from both simulated and real data, and searching the (*q_h_, q*) parameter space for regions that minimise the distance between the summary statistics from the simulated and real data.

We used 33 summary statistics (captured at endemic equilibrium) in the calibration process:

-overall prevalence in the population (with weight 1),

-prevalence in 16 age groups (with weights as the percentage of people in the given population groups),

-prevalence in 12 household size groups (with weights as the percentage of people in the given population groups),

-percentage of households with zero, at least one, at least two, at least three cases (with weights as 0.25 summing to 1).

These statistics are collected from a new generated sample quarterly for five years (20 times total) in every simulation run. To match the sample characteristics of the data, a sample with size 1300 is selected uniformly at random from 480 houses selected uniformly at random. We used logarithm of Euclidian distance as the distance function.

The result of the model calibration is shown in **Fig. S*5***. In this plot, darker regions indicate a greater belief in the true values of the parameters *q_h_* and *q.* We simulated the model using 100 values of the *q* & *q_h_* parameters generated from the posterior distribution (red points in **Fig. S*6***). The overall prevalence, prevalence in age and household groups are compared with the real data in **Fig. S*7***.


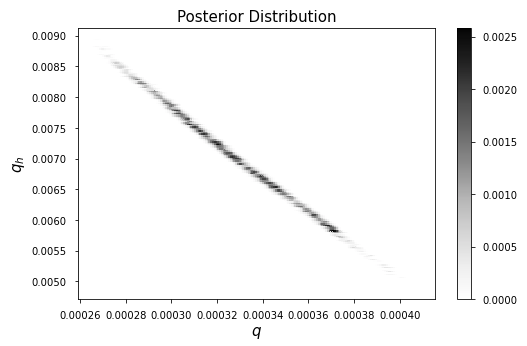


**Fig. S5:** Marginal posterior distribution for the household and community transmission coefficients (q_h_ and q) estimated using BOLFI


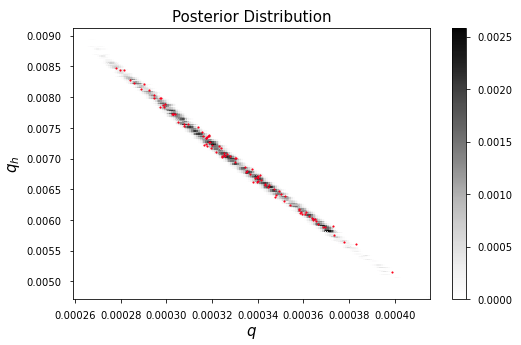


**Fig. S6:** Marginal posterior distribution for the household and community transmission coefficients (q_h_ and q) and sampled points from posterior (red).


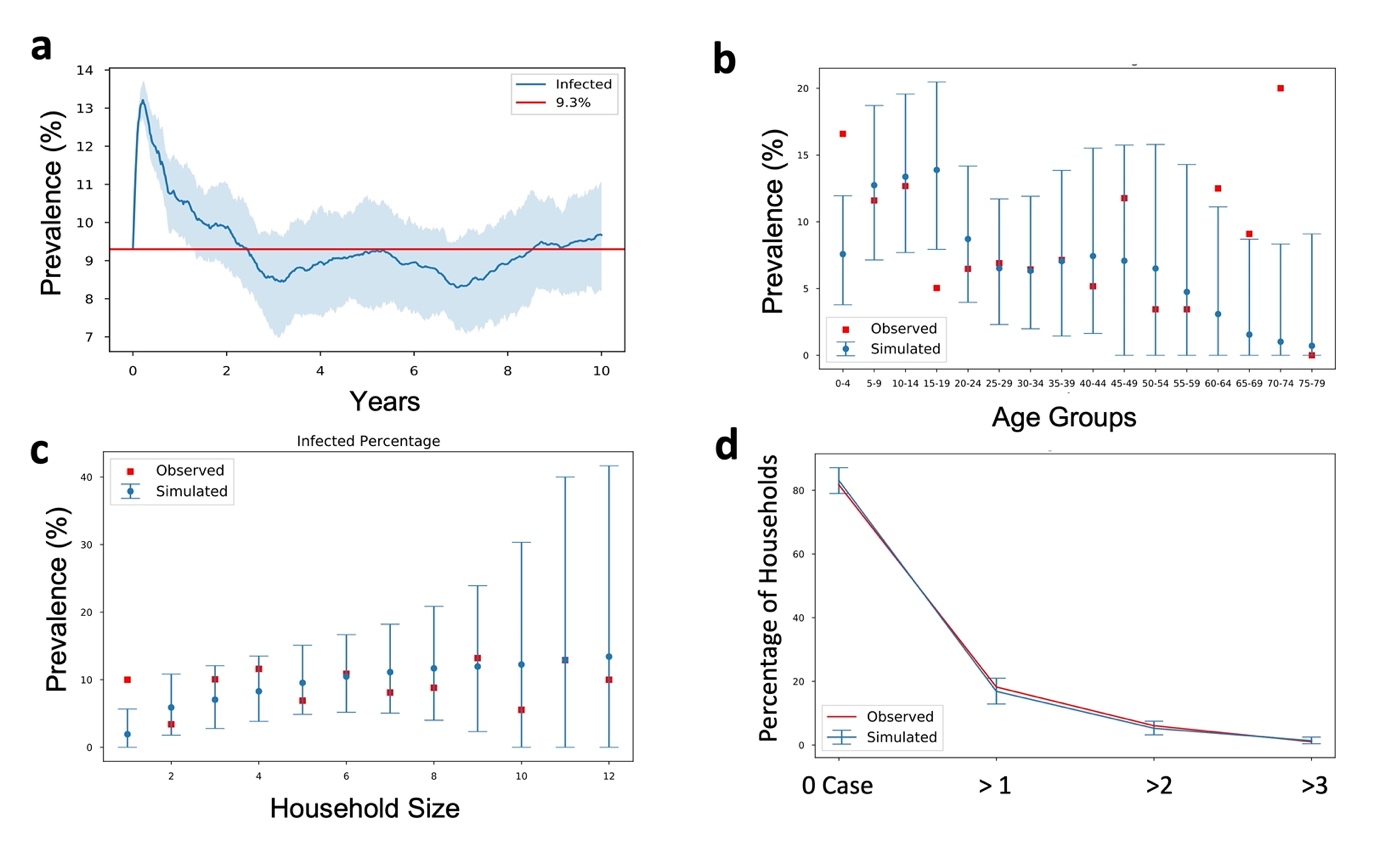


**Fig. S7:** (a) Mean and 2.5–97.5% quantiles of overall prevalence, (b) mean and 2.5–97.5% quantiles of prevalence in age groups, (c) mean and 2.5–97.5% quantiles of prevalence in household groups, (d) mean and 2.5–97.5% quantiles of the percentage of household groups with zero, at least one, at least two, at least three cases. In panels, blue represents simulation results and red represents the survey data. Data is captured quarterly after year 5 in panes (b), (c), and (d).

Using the calibrated model, we next looked at the community transmission percentage, i.e., the percentage of all transmission events which occur outside of households. The calibrated model shows that community transmission is responsible for an average of 50% of cases with 2.5–97.5% quantiles of [42%, 59%] of all the community and household transmissions (**Fig. S*8***). While the average community transmission percentage of people betwe en ages of 5–19 is 60%, the percentage decreases in older age groups suggesting that young people are more likely to introduce scabies into households. The percentage of infestations acquired in households increases from 0% to 50% as household size increases from one to five members, stabilising at 50% thereafter, suggesting that larger households do not necessarily have higher household transmission. Note that quantiles are very wide among older age groups and very large households as these occur less commonly in the simulated populations. We observed that the community transmission percentage was quite sensitive to the precise combination of *q* and *q_h_* values used.


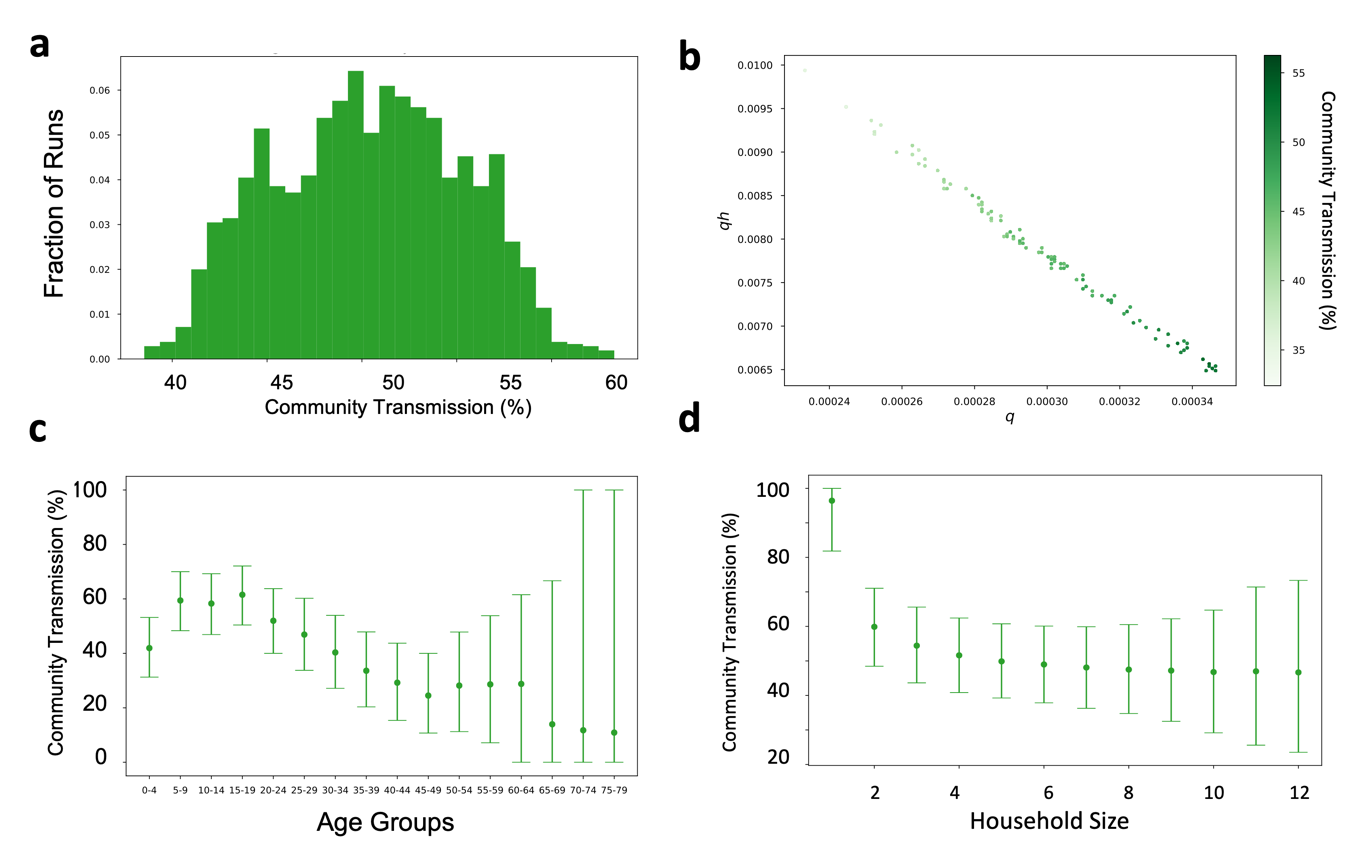


**Fig. S8:** (a) Histogram of overall community transmission percentage, (b) distribution of overall community transmission percentage, (c) mean and 2.5–97.5% quantiles of community transmission percentages in age groups and (d) mean and 2.5–97.5% quantiles of community transmission percentages in household size groups. Data is captured quarterly after year 5 in 100 simulations.

# **S3. R_0_ Estimation**

We have estimated R_0_ by running simulations. We initialized the simulation with one person having scabies and simulated the epidemic for 2 years (5 years) for scenarios with 90 days (150 days) duration of infestation. We run the simulation 10000 times (1000 times for each combination of *q* and *q_h_* parameters).

As only the source ID of household transmission is recorded in our modelling framework, we first estimated the secondary household infestations from the first person infested in every household in the first year (**Algorithm *1***). Then we estimated the secondary infections rate by dividing secondary household infections rate by household transmission percentage and we estimated R_0_ by taking the mean of all the secondary infections rate.

**Algorithm 1:** Pseudo code of R_0_ estimation

secondary_hh_infections_list = []

for every simulation run:

if no transmission occurred:

Add 0 to secondary_hh_infections_list

else:

if there is at least one community transmission:

if the first transmission is in the household:

Add the number of household infections from the first infected seed

for every other person, agent i infected in the community and brought scabies to their households for the first time:

Add the number of household infections from agent i

else: #there is only hh transmission

Add the number of household infections from the first infected seed in the first year

Calculate household transmission percentage among all the transmission events

Calculate all secondary infections by dividing every item in secondary_hh_infections_list by household transmission percentage

Calculate the mean and quantiles of all secondary infections

We estimated R_0_ in scenarios with baseline duration of infestation and reduced duration of infestations with average of 90 days and 150 days (**Table S2**).

**Table S2:** Estimation of R_0_ in baseline and other scenarios with lower durations of infestation. R_0_ estimations calculated using baseline duration of infestation and reduced duration of infestation are presented for simulations with average of 90 days and 150 days duration of infestation.

|  | Baseline | | 10% Reduction | | 20% Reduction | |
| --- | --- | --- | --- | --- | --- | --- |
|  | **90 days** | **150 days** | **81 days** | **135 days** | **72 days** | **120 days** |
| R_0_ | 1.24 | 1.237 | 1.107 | 1.18 | 0.989 | 1.04 |

# **S4. Estimation of disability-adjusted life-years (DALYs)**

We summed the duration of infestation for every infested person in 20 years to estimate the total person-years with scabies for 20 years starting from the beginning of the first MDA round. After calculating the mean and quantiles of the total person-years, we estimated the mean and quantiles of DALYs by multiplying the mean and quantiles of the total person-years by the disability weight assigned to scabies, 0.027 [9]. We then divided DALYs estimations for the entire population by “the average population size in 20 years/10,000” to estimate DALYs per 10,000 people.

In order to calculate DALYs averted, we estimated DALYs for every run for the “No MDA” scenario. Then, we subtracted DALYs of MDA scenarios from DALYs of “No MDA” scenarios for each parameter set. Then, we calculated DALYs averted by taking the mean and quantiles of obtained results.

For discounted DALYs averted estimation, we estimated yearly DALYs for every run for the “No MDA” and “MDA scenarios and multiplied them by the corresponding discount factor. We calculated discount factors as 1, 0.971, 0.943, 0.915,… for t = 0,1,2,3… using the formulation of 1/(1+r)^t with a discount fraction of r = 0.03. Then, we subtracted discounted DALYs of MDA scenarios from discounted DALYs of “No MDA” scenarios for each parameter set. Then, we calculated discounted DALYs averted by taking the mean and quantiles of obtained results.

# **S5. Sensitivity analysis of duration of infestation**

We conducted a sensitivity analysis around the average infestation days. We set the average duration of infestation as 150 days rather than 90 days. We first calibrated the parameter set (*q* and *q_h_*) using 150 days by using BOLFI. We ended up with quite similar age- and household-specific scabies prevalence in the calibrated model (**Fig. S*9*** and **Fig. S*10***) as model calibration decreased *q* and *q_h_* values in accordance with the increase in the duration of infestation (**Fig. S*10***.b).


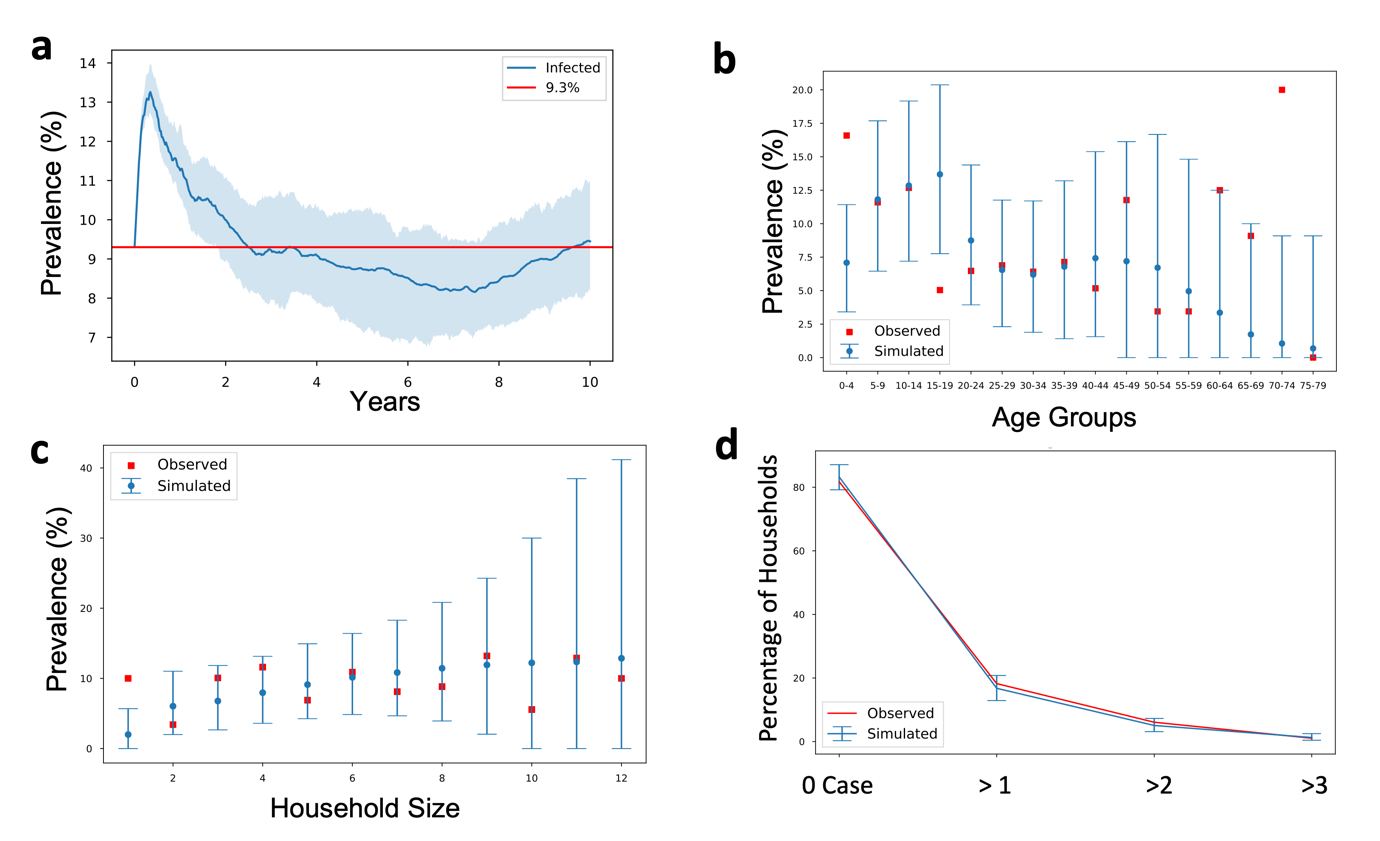


**Fig. S9**: (a) Mean and 2.5–97.5% quantiles of overall prevalence, (b) mean and 2.5–97.5% quantiles of prevalence in age groups, (c) mean and 2.5–97.5% quantiles of prevalence in household groups, (d) mean and 2.5–97.5% quantiles of the percentage of household groups with zero, at least one, at least two, at least three cases **with an average of 150 days of infestation duration**. In panels, blue represents simulation results and red represents the survey data. Data is captured quarterly after year 5 in panels (b), (c), and (d).


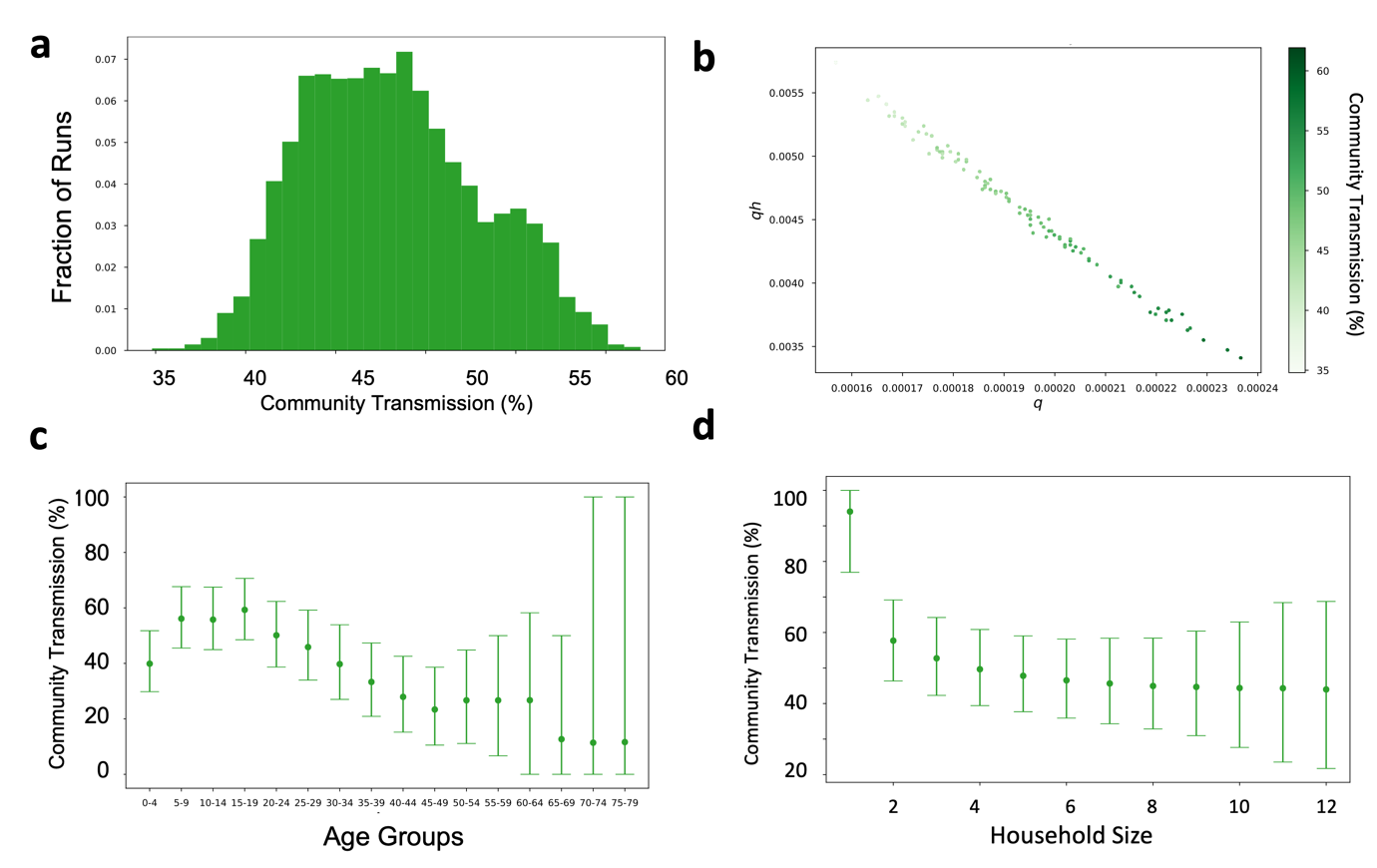


**Fig. S10:** (a) Histogram of overall community transmission percentage, (b) distribution of overall community transmission percentage, (c) mean and 2.5–97.5% quantiles of community transmission percentages in age groups and (d) mean and 2.5–97.5% quantiles of community transmission percentages in household size groups **with an average of 150 days of infestation duration**. Data is captured quarterly after year 5 in 100 simulations.

Even though decreasing the transmission parameters proportionate to the increase in the duration of infestation was necessary to keep the endemic prevalence or R_0_ at the same level, the decrease in the transmission parameters makes a difference when it comes to interventions (Fig. **S11**). When the transmission coefficients are lower (higher duration of infestation), the disease spreads more slowly in the community. Therefore, repetitive MDA rounds became more effective under the assumption of lower transmission coefficients (higher duration of infestation) (Fig. **S12**, Fig. **S13**, Table S3, and Table S4).
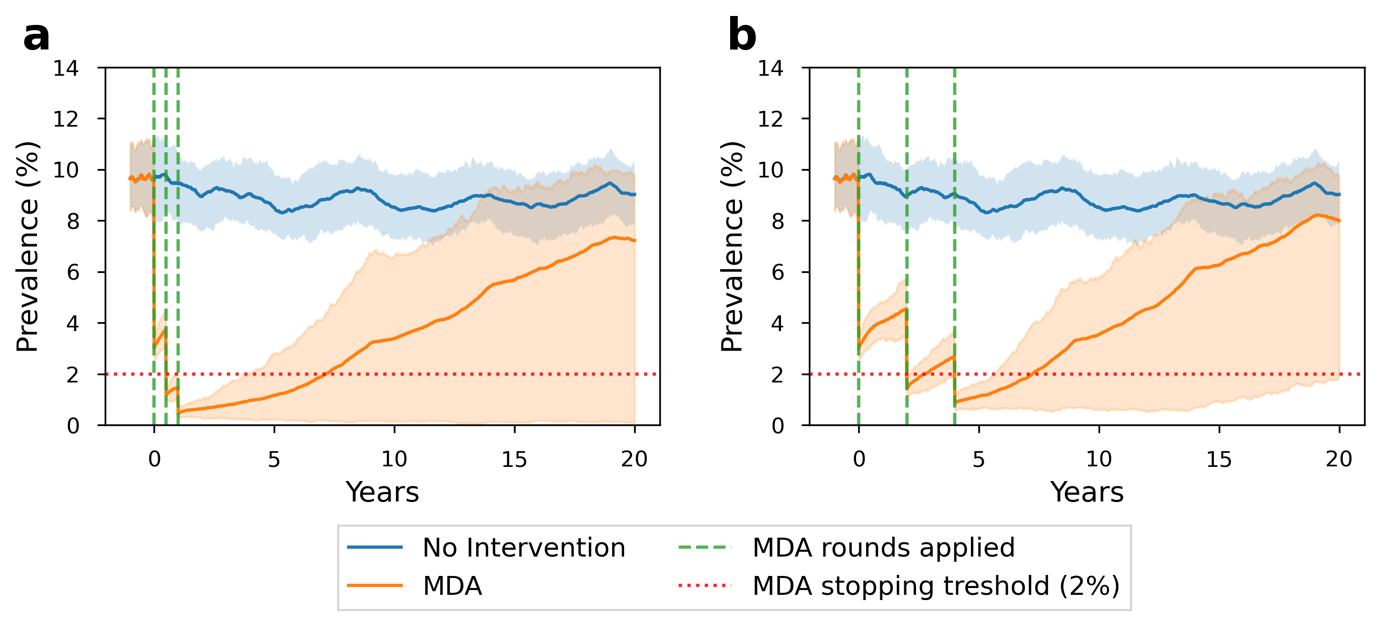


**Fig. S11:** The scabies prevalence in 20 years with MDA strategies consisting of 3 rounds, 80% population coverage, random individual selection, and (a) six-month (b) two-year time intervals **with an average of duration of infestation of 150 days**. The green dashed lines show when MDA rounds are applied. The red dotted lines represent MDA stopping threshold (2%). It is assumed that there is no scabies importation.

**Fig. S12:** The proportion of simulation runs with less than 2% prevalence is achieved in differing MDA strategies **with an average of duration of infestation of 150 days**. The first column (a & c) are the MDA strategies with household-based selection and the second column (b & d) are the MDA strategies with random individual selection. The first row (a & b) are the MDA strategies with three rounds and the second row (c & d) are the MDA strategies with 5 rounds. Each panel is grouped by the population coverage in MDAs and MDA intervals. Each value is calculated from 100 simulation runs. In these scenarios, it is assumed that there is no scabies importation.

**Fig. S13:** Mean and 2.5–97.5 quantiles of DALYs averted per 10000 people in differing MDA strategies **with an average duration of infestation of 150 days**. The values are calculated in 20 years starting from the first MDA round. The first column (a & c) are the MDA strategies with household-based selection and the second column (b & d) are the MDA strategies with random individual selection. The first row (a & b) are the MDA strategies with three rounds and the second row (c & d) are the MDA strategies with 5 rounds. Each panel is grouped by the population coverage in MDAs and MDA intervals. Each value is calculated from 100 simulation runs. In these scenarios, it is assumed that there is no scabies importation.

**Table S3:** Percentage of simulations with <=2% prevalence 1 year after the last MDA round, percentage of simulations with scabies elimination, DALYs averted per 10,000 people (mean, 2.5–97.5 quantiles), and time until prevalence returns to baseline are presented in **MDA strategies with 3 rounds with an average duration of infestation of 150 days**. Time until prevalence returns to baseline is calculated among the runs in which scabies is not eliminated. It is assumed that there is no scabies importation. MDA strategies are ordered from best to worst in terms of (undiscounted) DALYs averted.

| Population selection | Population coverage (%) | MDA Interval | The percentage of runs with <=2% prevalence 1 year after the last MDA round (%) | Percentage of runs with scabies elimination  (%) | DALYs averted per 10,000 people in 20 years  (Mean, 2.5 – 97.5 quantiles) | Discounted DALYs averted per 10,000 people in 20 years  (Mean, 2.5 – 97.5 quantiles) | Time until prevalence returns to baseline  (years) |
| --- | --- | --- | --- | --- | --- | --- | --- |
| Household | 90 | 6 months | 100 | 0 | 352.2 (221.5, 470.2) | 279.1 (189.2, 357.9) | 20+ (15.9, 20+) |
| Random | 90 | 6 months | 100 | 0 | 343.6 (216.2, 467.7) | 273.4 (185.6, 354.9) | 20+ (16.0, 20+) |
| Household | 90 | 1 year | 100 | 0 | 335.7 (215.8, 461.9) | 267.2 (188.0, 350.0) | 20+ (15.4, 20+) |
| Random | 90 | 1 year | 100 | 0 | 327.7 (209.2, 466.5) | 261.6 (181.0, 352.2) | 20+ (14.1, 20+) |
| Household | 90 | 2 years | 100 | 0 | 322.7 (225.5, 447.9) | 255.6 (188.3, 337.2) | 20+ (15.6, 20+) |
| Random | 90 | 2 years | 100 | 0 | 316.5 (199.2, 449.4) | 251.0 (171.7, 338.9) | 20+ (15.4, 20+) |
| Household | 80 | 6 months | 100 | 0 | 296.5 (175.5, 454.4) | 239.9 (156.3, 344.7) | 20+ (13.0, 20+) |
| Household | 80 | 1 year | 100 | 0 | 272.8 (165.3, 440.6) | 222.0 (146.4, 333.6) | 20+ (13.0, 20+) |
| Random | 80 | 6 months | 100 | 0 | 269.1 (153.6, 450.9) | 220.5 (138.9, 345.0) | 20+ (12.7, 20+) |
| Random | 80 | 1 year | 100 | 0 | 255.2 (153.0, 439.0) | 209.4 (136.8, 332.4) | 20+ (12.7, 20+) |
| Household | 80 | 2 years | 100 | 0 | 242.2 (166.4, 396.2) | 198.0 (143.6, 304.3) | 18.9 (13.1, 20+) |
| Random | 80 | 2 years | 100 | 0 | 234.5 (151.1, 363.2) | 192.4 (133.4, 277.3) | 18.7 (13.2, 20+) |
| Household | 70 | 6 months | 100 | 0 | 208.4 (128.1, 408.1) | 176.1 (115.9, 308.6) | 17.9 (11.3, 20+) |
| Random | 70 | 6 months | 97 | 0 | 192.1 (117.7, 296.6) | 164.1 (108.3, 241.3) | 17.0 (11.1, 20+) |
| Household | 70 | 1 year | 92 | 0 | 186.2 (118.0, 348.8) | 158.3 (108.7, 270.4) | 17.1 (11.4, 20+) |
| Household | 70 | 2 years | 66 | 0 | 183.2 (118.2, 276.2) | 153.8 (108.1, 221.0) | 17.2 (11.4, 20+) |
| Random | 70 | 1 year | 90 | 0 | 182 (115.7, 292.9) | 155.3 (106.5, 231.8) | 16.4 (12.4, 20+) |
| Random | 70 | 2 years | 53 | 0 | 172 (115.0, 233.4) | 145.5 (102.7, 190.8) | 16.3 (12.3, 20+) |
| Household | 60 | 6 months | 69 | 0 | 147.8 (88.0, 225.5) | 128.9 (83.4, 190.4) | 13.6 (8.3, 20+) |
| Household | 60 | 1 year | 30 | 0 | 140.8 (96.6, 205.7) | 122.6 (88.4, 170.9) | 13.4 (8.3, 20+) |
| Household | 60 | 2 years | 6 | 0 | 134.3 (87.8, 186.0) | 115.2 (80.4, 153.9) | 13.4 (9.3, 20+) |
| Random | 60 | 1 year | 11 | 0 | 133.1 (84.8, 205.2) | 116.2 (79.0, 167.5) | 13.3 (8.2, 20+) |
| Random | 60 | 2 years | 1 | 0 | 127.4 (80.9, 188.9) | 109.6 (73.7, 153.9) | 13.3 (8.7, 20+) |
| Random | 60 | 6 months | 43 | 0 | 105.2 (49.0, 152.9) | 96.3 (50.2, 134.2) | 9.7 (6.7, 20+) |

**Table S4:** Percentage of simulations with <=2% prevalence 1 year after the last MDA round, percentage of simulations with scabies elimination, DALYs averted per 10,000 people (mean, 2.5–97.5 quantiles), and time until prevalence returns to baseline are presented in **MDA strategies with 5 rounds with an average of duration of infestation of 150 days.** Time until prevalence returns to baseline is calculated among the runs in which scabies is not eliminated. It is assumed that there is no scabies importation. MDA strategies are ordered from best to worst in terms of (undiscounted) DALYs averted.

| Population selection | Population coverage (%) | MDA Interval | The percentage of runs with <=2% prevalence 1 year after the last MDA round (%) | Percentage of runs with scabies elimination  (%) | DALYs averted per 10,000 people in 20 years  (Mean, 2.5 – 97.5 quantiles) | Discounted DALYs averted per 10,000 people in 20 years  (Mean, 2.5 – 97.5 quantiles) | Time until prevalence returns to baseline  (years) |
| --- | --- | --- | --- | --- | --- | --- | --- |
| Random | 90 | 2 years | 100 | 1 | 411.2 (330.2, 464.3) | 313.3 (262.2, 349.3) | 20+ (20.0, 20+) |
| Household | 90 | 2 years | 100 | 2 | 410.1 (347.4, 460.4) | 312.9 (271.0, 347.6) | 20+ (20.0, 20+) |
| Household | 90 | 6 months | 100 | 12 | 393.3 (275.4, 485.4) | 307.3 (229.1, 366.5) | 20+ (17.9, 20+) |
| Random | 90 | 6 months | 100 | 9 | 390.7 (261.7, 484.6) | 305.4 (221.6, 370.0) | 20+ (16.8, 20+) |
| Household | 90 | 1 year | 100 | 9 | 390 (285.2, 476.2) | 303.9 (238.0, 361.6) | 20+ (17.8, 20+) |
| Random | 90 | 1 year | 100 | 3 | 388.2 (280.8, 471.6) | 302.6 (231.8, 358.6) | 20+ (17.7, 20+) |
| Random | 80 | 6 months | 100 | 0 | 374.7 (257.8, 471.1) | 294.3 (216.1, 358.8) | 20+ (17.5, 20+) |
| Household | 80 | 6 months | 100 | 1 | 373.6 (244.3, 472.5) | 293.4 (208.4, 356.7) | 20+ (16.0, 20+) |
| Random | 80 | 2 years | 100 | 1 | 372.8 (294.1, 444.3) | 285.2 (235.4, 332.8) | 20+ (18.7, 20+) |
| Household | 80 | 1 year | 100 | 1 | 371.9 (265.4, 465.7) | 290.5 (221.0, 351.4) | 20+ (17.8, 20+) |
| Household | 80 | 2 years | 100 | 0 | 371.1 (287.4, 444.3) | 284.6 (227.1, 331.9) | 20+ (19.8, 20+) |
| Random | 80 | 1 year | 100 | 2 | 365.7 (262.6, 462.0) | 286.3 (217.4, 349.2) | 20+ (16.4, 20+) |
| Household | 70 | 6 months | 100 | 0 | 352.6 (225.0, 464.7) | 278.5 (194.4, 352.5) | 20+ (14.0, 20+) |
| Random | 70 | 6 months | 100 | 1 | 351.3 (235.7, 468.2) | 277.5 (199.0, 354.5) | 20+ (16.6, 20+) |
| Household | 70 | 1 year | 100 | 1 | 343.8 (224.3, 456.6) | 269.8 (190.8, 342.8) | 20+ (16.6, 20+) |
| Random | 70 | 1 year | 100 | 0 | 333.8 (201.0, 455.9) | 262.8 (172.8, 343.3) | 20+ (14.8, 20+) |
| Household | 70 | 2 years | 98 | 0 | 316.5 (234.0, 422.5) | 245.3 (188.1, 316.1) | 20+ (16.7, 20+) |
| Random | 70 | 2 years | 94 | 0 | 308.5 (212.9, 416.3) | 239.4 (172.4, 310.5) | 20+ (17.0, 20+) |
| Household | 60 | 6 months | 100 | 0 | 289.7 (179.2, 443.9) | 233.9 (155.1, 336.4) | 20+ (13.3, 20+) |
| Household | 60 | 1 year | 100 | 0 | 275.5 (169.5, 431.1) | 221.3 (147.0, 324.4) | 20+ (13.7, 20+) |
| Random | 60 | 6 months | 100 | 0 | 271.3 (171.2, 454.5) | 220.7 (151.0, 344.2) | 20+ (12.8, 20+) |
| Random | 60 | 1 year | 100 | 0 | 261.5 (158.1, 426.2) | 210.9 (137.1, 322.9) | 20+ (13.2, 20+) |
| Household | 60 | 2 years | 55 | 0 | 243.1 (166.7, 335.7) | 192.5 (140.2, 253.0) | 20+ (14.8, 20+) |
| Random | 60 | 2 years | 48 | 0 | 228.9 (159.2, 370.0) | 182.2 (133.1, 275.6) | 20+ (15.2, 20+) |

# **S6. Additional MDA results**

In this section, we present DALYs averted heatmap plot (**Fig. S*14***) and tables containing the results of all the MDA scenarios including the ones with 60% and 70% population coverage (**Table S*5*** and **Table S*6***). In section S6.1, we provide results with treatment efficacies of 85% (Section S6.1.1) and 95% (Section S.1.2).

**Fig. S14:** Mean and 2.5–97.5 quantiles of DALYs averted per 10000 people in differing MDA strategies with **an average duration of infestation of 90 days**. The values are calculated in 20 years starting from the first MDA round. The first column (a & c) are the MDA strategies with household-based selection and the second column (b & d) are the MDA strategies with random individual selection. The first row (a & b) are the MDA strategies with three rounds and the second row (c & d) are the MDA strategies with 5 rounds. Each panel is grouped by the population coverage in MDAs and MDA intervals. Each value is calculated from 100 simulation runs. In these scenarios, it is assumed that there is no scabies importation.

**Table S5:** Percentage of simulations with <=2% prevalence 1 year after the last MDA round, percentage of simulations with scabies elimination, DALYs averted per 10,000 people (mean, 2.5–97.5 quantiles), and time until prevalence returns to baseline are presented in **MDA strategies with 3 rounds in scenarios with an average duration of infestation of 90 days**. Time until prevalence returns to baseline is calculated among the runs in which scabies is not eliminated. It is assumed that there is no scabies importation. MDA strategies are ordered from best to worst in terms of (undiscounted) DALYs averted.

| Population selection | Population coverage (%) | MDA Interval | The percentage of runs with <=2% prevalence 1 year after the last MDA round (%) | Percentage of runs with scabies elimination  (%) | DALYs averted per 10,000 people in 20 years  (Mean, 2.5 – 97.5 quantiles) | Discounted DALYs averted per 10,000 people in 20 years  (Mean, 2.5 – 97.5 quantiles) | Time until prevalence returns to baseline  (years) |
| --- | --- | --- | --- | --- | --- | --- | --- |
| Household | 90 | 6 months | 100 | 0 | 218.2 (91.1, 459.0) | 183.9 (88.0, 347.6) | 18.4 (8.2, 20+) |
| Random | 90 | 6 months | 100 | 0 | 210.6 (106.4, 450.2) | 178.8 (103.1, 340.5) | 17.7 (7.7, 20+) |
| Household | 90 | 1 year | 100 | 0 | 203.6 (112.3, 433.3) | 172.3 (104.7, 328.6) | 17.7 (8.1, 20+) |
| Random | 90 | 1 year | 100 | 0 | 188.6 (108.6, 441.9) | 161.8 (101.0, 334.8) | 13.6 (8.1, 20+) |
| Random | 90 | 2 years | 91 | 0 | 168.5 (108.5, 367.4) | 144.9 (100.8, 280.5) | 13.2 (8.5, 20+) |
| Household | 90 | 2 years | 92 | 0 | 159.3 (108.0, 243.6) | 138.9 (101.2, 203.0) | 12.8 (8.7, 17.8) |
| Household | 80 | 6 months | 99 | 0 | 143.9 (91.0, 267.5) | 128.3 (84.9, 220.8) | 12.3 (6.9, 17.9) |
| Random | 80 | 6 months | 100 | 0 | 134.3 (81.5, 237.0) | 120.3 (76.0, 201.4) | 12.2 (6.8, 18.4) |
| Household | 80 | 1 year | 78 | 0 | 127.2 (80.4, 208.8) | 113.9 (77.4, 178.2) | 11.8 (7.1, 13.8) |
| Random | 80 | 1 year | 76 | 0 | 127.1 (76.1, 184.2) | 113.6 (73.2, 160.3) | 11.2 (7.2, 20+) |
| Household | 80 | 2 years | 35 | 0 | 121.3 (80.1, 175.7) | 107.6 (75.0, 148.8) | 11.4 (8.0, 19.0) |
| Random | 80 | 2 years | 26 | 0 | 117.6 (82.6, 174.9) | 104.3 (77.7, 148.8) | 11.4 (7.6, 17.9) |
| Household | 70 | 1 year | 21 | 0 | 100.2 (56.8, 143.7) | 91.1 (56.5, 127.2) | 8.2 (6.4, 13.4) |
| Household | 70 | 2 years | 2 | 0 | 99.2 (67.3, 139.6) | 88.4 (61.6, 118.8) | 8.8 (7.6, 13.5) |
| Household | 70 | 6 months | 69 | 0 | 97.1 (60.1, 146.0) | 89.5 (59.3, 128.7) | 8.0 (6.2, 13.3) |
| Random | 70 | 6 months | 58 | 0 | 96.5 (54.6, 142.6) | 89.0 (56.7, 126.4) | 8.0 (6.2, 13.2) |
| Random | 70 | 1 year | 12 | 0 | 93.9 (45.3, 129.0) | 85.7 (48.8, 113.6) | 8.0 (6.5, 13.9) |
| Random | 70 | 2 years | 0 | 0 | 92 (54.8, 125.6) | 82.5 (54.2, 108.9) | 8.6 (7.3, 13.2) |
| Household | 60 | 6 months | 16 | 0 | 80.5 (44.6, 116.8) | 74.3 (46.1, 103.6) | 7.5 (5.6, 13.5) |
| Household | 60 | 1 year | 0 | 0 | 77.7 (39.3, 119.2) | 71.0 (40.7, 103.5) | 7.5 (5.7, 13.7) |
| Household | 60 | 2 years | 0 | 0 | 76.6 (39.1, 117.3) | 68.6 (42.0, 100.1) | 8.1 (1.5, 13.8) |
| Random | 60 | 1 year | 0 | 0 | 75.3 (36.8, 109.3) | 68.8 (40.0, 94.9) | 7.3 (5.6, 13.4) |
| Random | 60 | 6 months | 4 | 0 | 73.6 (32.6, 107.0) | 68.6 (34.5, 96.2) | 7.1 (5.7, 13.0) |
| Random | 60 | 2 years | 0 | 0 | 71.5 (40.9, 104.6) | 64.2 (39.7, 88.7) | 7.9 (1.5, 13.3) |

**Table S6:** Percentage of simulations with <=2% prevalence 1 year after the last MDA round, percentage of simulations with scabies elimination, DALYs averted per 10,000 people (mean, 2.5–97.5 quantiles), and time until prevalence returns to baseline are presented in **MDA strategies with 5 rounds in scenarios with an average duration of infestation of 90 days**. Time until prevalence returns to baseline is calculated among the runs in which scabies is not eliminated. It is assumed that there is no scabies importation. MDA strategies are ordered from best to worst in terms of (undiscounted) DALYs averted.

| Population selection | Population coverage (%) | MDA Interval | The percentage of runs with <=2% prevalence 1 year after the last MDA round (%) | Percentage of runs with scabies elimination  (%) | DALYs averted per 10,000 people in 20 years  (Mean, 2.5 – 97.5 quantiles) | Discounted DALYs averted per 10,000 people in 20 years  (Mean, 2.5 – 97.5 quantiles) | Time until prevalence returns to baseline  (years) |
| --- | --- | --- | --- | --- | --- | --- | --- |
| Household | 90 | 6 months | 100 | 18 | 341.7 (161.7, 487.4) | 271.1 (144.7, 369.3) | 20+ (11.6, 20+) |
| Random | 90 | 1 year | 100 | 10 | 330.8 (204.2, 477.5) | 262.4 (175.2, 359.4) | 20+ (12.7, 20+) |
| Household | 90 | 1 year | 100 | 10 | 326.1 (191.5, 462.4) | 259.7 (166.7, 350.8) | 20+ (12.7, 20+) |
| Random | 90 | 6 months | 100 | 16 | 325 (164.2, 488.9) | 259.9 (146.8, 370.8) | 20+ (11.7, 20+) |
| Household | 90 | 2 years | 99 | 0 | 307.5 (210.1, 429.2) | 242.0 (176.3, 320.7) | 20+ (13.8, 20+) |
| Random | 90 | 2 years | 98 | 3 | 306.2 (202.0, 437.7) | 241.0 (171.4, 329.8) | 20+ (13.5, 20+) |
| Random | 80 | 6 months | 100 | 0 | 298 (144.7, 461.8) | 240.7 (132.9, 350.7) | 20+ (11.2, 20+) |
| Household | 80 | 6 months | 100 | 0 | 283.4 (158.1, 458.3) | 231.6 (142.3, 347.6) | 20+ (11.6, 20+) |
| Household | 80 | 1 year | 100 | 0 | 269.8 (165.5, 458.6) | 219.5 (147.7, 346.9) | 20+ (11.5, 20+) |
| Random | 80 | 1 year | 100 | 0 | 248.3 (150.0, 419.0) | 205.0 (135.4, 316.8) | 18.2 (11.7, 20+) |
| Random | 70 | 6 months | 100 | 0 | 228.8 (120.2, 448.6) | 191.4 (112.2, 337.3) | 18.0 (8.7, 20+) |
| Household | 70 | 6 months | 100 | 0 | 228 (133.0, 441.1) | 190.6 (120.9, 333.0) | 18.2 (8.5, 20+) |
| Household | 80 | 2 years | 64 | 0 | 212.5 (156.6, 291.7) | 175.2 (132.6, 231.7) | 16.4 (12.9, 20+) |
| Random | 80 | 2 years | 46 | 0 | 197.1 (144.6, 290.2) | 163.8 (125.9, 231.9) | 15.9 (12.4, 20+) |
| Household | 70 | 1 year | 97 | 0 | 193 (123.8, 381.9) | 164.2 (111.1, 295.3) | 13.7 (10.8, 20+) |
| Random | 70 | 1 year | 98 | 0 | 174.8 (112.2, 409.9) | 150.2 (102.2, 308.8) | 13.2 (8.6, 20+) |
| Household | 70 | 2 years | 6 | 0 | 160.3 (121.0, 209.1) | 135.2 (105.7, 172.8) | 13.5 (11.8, 18.3) |
| Household | 60 | 6 months | 98 | 0 | 156.9 (87.0, 345.2) | 137.1 (82.2, 272.4) | 12.9 (7.8, 20+) |
| Random | 70 | 2 years | 3 | 0 | 150.9 (106.9, 197.7) | 127.8 (94.0, 163.3) | 13.3 (11.9, 17.6) |
| Random | 60 | 6 months | 91 | 0 | 140.7 (86.0, 245.0) | 124.6 (83.7, 202.1) | 12.3 (7.2, 18.9) |
| Household | 60 | 1 year | 67 | 0 | 134.6 (95.2, 180.7) | 118.8 (88.5, 155.6) | 12.2 (8.4, 17.7) |
| Random | 60 | 1 year | 38 | 0 | 129.5 (83.6, 181.3) | 113.8 (79.3, 153.6) | 12.1 (8.0, 18.4) |
| Household | 60 | 2 years | 0 | 0 | 125.5 (89.1, 163.6) | 106.6 (78.9, 135.4) | 12.9 (1.5, 17.7) |
| Random | 60 | 2 years | 0 | 0 | 117.2 (84.5, 156.1) | 99.7 (72.7, 128.5) | 12.8 (1.5, 18.7) |

## **S6.1. Results with various treatment efficacies**

In the main manuscript, we present results of MDA strategies with 90% treatment efficacy. In this section, we present the comparison of MDA strategies under the assumption of 85% treatment efficacy (Section S6.1.1) and 95% treatment efficacy (Section S6.1.2). In Section S6.1.3, we show how people not receiving one or two doses in each round impact efficacy of an MDA strategy and how this can be explained through lower treatment efficacy and population coverage.

### **S6.1.1. Results with treatment efficacy of 85% (rather than 90%)**

In MDA strategies with treatment efficacy of 85% (rather than 90%), 80% population coverage, household selection in 5 rounds with 2-year time interval, the probability of achieving a prevalence of less than the stopping threshold decreases from 0.64 to 0.24 (**Fig. S*15***). Average DALYs averted per 10,000 people in 20 years decreases from 80.5 years to 75 years in MDA strategies with 60% coverage, household-based individual selection in 3 rounds with 6-month time interval when a treatment efficacy of 85% is assumed rather than 90% (**Table S*7***). The probability of scabies elimination decreases from 18% to 5% in MDA strategies with 90% coverage, household-based individual selection in 5 rounds with 6-month time interval when a treatment efficacy of 85% is assumed rather than 90% (**Table S*8***).

**Fig. S15:** The proportion of simulation runs with a prevalence of less than 2% is achieved in differing MDA strategies having a treatment efficacy of 85% (rather than 90%) in scenarios with **an average duration of infestation of 90 days**. The first column (a & c) are the MDA strategies with household-based selection and the second column (b & d) are the MDA strategies with random individual selection. The first row (a & b) are the MDA strategies with three rounds and the second row (c & d) are the MDA strategies with 5 rounds. Each panel is grouped by the population coverage in MDAs and MDA intervals. Each value is calculated from 100 simulation runs. It is assumed that there is no scabies importation.

**Table S7:** Percentage of simulations with <=2% prevalence 1 year after the last MDA round, percentage of simulations with scabies elimination, DALYs averted per 10,000 people (mean, 2.5–97.5 quantiles), and time until prevalence returns to baseline are presented in **MDA strategies with 3 rounds and 85% treatment efficacy**  in scenarios with **an average duration of infestation of 90 days.** Time until prevalence returns to baseline is calculated among the runs in which scabies is not eliminated. It is assumed that there is no scabies importation. MDA strategies are ordered from best to worst in terms of (undiscounted) DALYs averted.

| Population selection | Population coverage (%) | MDA Interval | The percentage of runs with <=2% prevalence 1 year after the last MDA round (%) | Percentage of runs with scabies elimination  (%) | DALYs averted per 10,000 people in 20 years  (Mean, 2.5 – 97.5 quantiles) | Discounted DALYs averted per 10,000 people in 20 years  (Mean, 2.5 – 97.5 quantiles) | Time until prevalence returns to baseline  (years) |
| --- | --- | --- | --- | --- | --- | --- | --- |
| Household | 90 | 6 months | 100 | 0 | 182.8 (89.1, 435.4) | 157.1 (86.5, 329.7) | 13.6 (7.6, 20.0) |
| Random | 90 | 6 months | 100 | 0 | 161.5 (92.9, 393.4) | 141.6 (88.1, 305.1) | 12.9 (7.2, 20.0) |
| Random | 90 | 1 year | 98 | 0 | 145.5 (92.3, 253.4) | 128.6 (86.2, 211.8) | 12.5 (7.6, 18.1) |
| Household | 90 | 1 year | 97 | 0 | 144 (97.2, 214.1) | 128.3 (91.0, 182.9) | 12.2 (7.7, 18.1) |
| Household | 90 | 2 years | 67 | 0 | 137.6 (96.5, 189.5) | 121.0 (88.7, 158.6) | 12.2 (8.1, 13.8) |
| Random | 90 | 2 years | 59 | 0 | 135.5 (92.0, 182.1) | 119.5 (87.6, 152.4) | 12.2 (7.8, 13.9) |
| Household | 80 | 6 months | 93 | 0 | 118.4 (79.7, 173.1) | 107.5 (77.0, 153.0) | 8.8 (6.9, 17.1) |
| Random | 80 | 6 months | 93 | 0 | 115 (64.5, 167.6) | 104.6 (65.7, 146.0) | 8.7 (6.6, 18.5) |
| Household | 80 | 1 year | 48 | 0 | 110 (69.7, 152.9) | 99.7 (69.5, 135.6) | 8.6 (6.6, 13.4) |
| Household | 80 | 2 years | 16 | 0 | 109.7 (75.5, 155.3) | 97.7 (70.8, 132.5) | 11.2 (7.8, 13.7) |
| Random | 80 | 1 year | 44 | 0 | 106.1 (70.9, 143.2) | 96.9 (70.3, 127.0) | 8.4 (6.6, 13.5) |
| Random | 80 | 2 years | 9 | 0 | 104.5 (68.2, 148.9) | 93.3 (66.0, 125.7) | 11.1 (7.5, 13.5) |
| Household | 70 | 6 months | 45 | 0 | 92.5 (50.2, 141.8) | 85.3 (52.4, 123.3) | 7.8 (6.2, 13.8) |
| Random | 70 | 6 months | 40 | 0 | 89.3 (53.0, 138.1) | 82.2 (55.2, 119.3) | 7.7 (5.9, 13.4) |
| Household | 70 | 2 years | 0 | 0 | 87.9 (54.6, 132.0) | 78.9 (51.6, 111.4) | 8.5 (1.7, 13.5) |
| Household | 70 | 1 year | 8 | 0 | 87.3 (50.6, 124.9) | 79.5 (51.1, 106.9) | 7.8 (6.1, 13.3) |
| Random | 70 | 1 year | 3 | 0 | 87.3 (49.7, 122.6) | 80.0 (52.3, 109.1) | 7.7 (6.4, 13.7) |
| Random | 70 | 2 years | 0 | 0 | 84 (53.1, 124.5) | 75.5 (52.3, 108.0) | 8.5 (1.6, 13.6) |
| Household | 60 | 6 months | 3 | 0 | 75 (40.9, 112.9) | 69.0 (41.7, 99.1) | 7.0 (5.1, 13.8) |
| Household | 60 | 1 year | 0 | 0 | 74.7 (34.8, 111.0) | 67.9 (38.2, 96.6) | 7.3 (5.8, 13.4) |
| Household | 60 | 2 years | 0 | 0 | 71.6 (36.9, 105.3) | 64.0 (36.8, 89.3) | 7.8 (1.5, 13.8) |
| Random | 60 | 1 year | 0 | 0 | 70.2 (34.3, 104.0) | 64.1 (36.1, 88.1) | 7.0 (5.4, 13.5) |
| Random | 60 | 6 months | 1 | 0 | 67.8 (35.2, 105.2) | 62.9 (38.6, 90.5) | 6.9 (4.4, 13.3) |
| Random | 60 | 2 years | 0 | 0 | 66.8 (32.9, 106.2) | 60.1 (35.0, 89.0) | 7.8 (1.5, 13.3) |

**Table S8:** Percentage of simulations with <=2% prevalence 1 year after the last MDA round, percentage of simulations with scabies elimination, DALYs averted per 10,000 people (mean, 2.5 – 97.5 quantiles), and time until prevalence returns to baseline are presented in **MDA strategies with 5 rounds and 85% treatment efficacy** in scenarios with **an average duration of infestation of 90 days.** Time until prevalence returns to baseline is calculated among the runs in which scabies is not eliminated. It is assumed that there is no scabies importation. MDA strategies are ordered from best to worst in terms of (undiscounted) DALYs averted.

| Population selection | Population coverage (%) | MDA Interval | The percentage of runs with <=2% prevalence 1 year after the last MDA round (%) | Percentage of runs with scabies elimination  (%) | DALYs averted per 10,000 people in 20 years  (Mean, 2.5 – 97.5 quantiles) | Discounted DALYs averted per 10,000 people in 20 years  (Mean, 2.5 – 97.5 quantiles) | Time until prevalence returns to baseline  (years) |
| --- | --- | --- | --- | --- | --- | --- | --- |
| Household | 90 | 6 months | 100 | 5 | 319.3 (157.4, 480.5) | 255.9 (140.4, 361.9) | 20+ (11.4, 20+) |
| Random | 90 | 6 months | 100 | 11 | 313.9 (159.4, 484.8) | 251.9 (141.1, 366.1) | 20+ (9.0, 20+) |
| Household | 90 | 1 year | 100 | 3 | 295.3 (184.9, 461.3) | 238.1 (162.5, 346.3) | 20+ (11.8, 20+) |
| Random | 90 | 1 year | 100 | 5 | 290.6 (175.0, 467.1) | 234.9 (152.2, 351.1) | 20+ (12.3, 20+) |
| Household | 80 | 6 months | 100 | 0 | 275.6 (144.8, 477.6) | 224.5 (129.0, 358.8) | 20+ (8.9, 20+) |
| Random | 80 | 6 months | 100 | 0 | 263.7 (115.8, 462.4) | 216.4 (105.6, 349.9) | 20+ (8.5, 20+) |
| Household | 90 | 2 years | 79 | 0 | 246.2 (178.6, 405.3) | 199.6 (152.0, 301.8) | 18.1 (13.3, 20+) |
| Random | 90 | 2 years | 80 | 0 | 241.3 (167.8, 384.6) | 196.1 (144.6, 287.8) | 17.9 (12.7, 20+) |
| Random | 80 | 1 year | 100 | 0 | 235.8 (141.7, 428.9) | 194.8 (127.1, 322.9) | 18.2 (11.1, 20+) |
| Household | 80 | 1 year | 99 | 0 | 225 (130.7, 413.9) | 187.6 (121.1, 310.7) | 17.7 (11.0, 20+) |
| Household | 70 | 6 months | 100 | 0 | 207 (112.0, 421.9) | 175.3 (103.4, 320.4) | 17.2 (8.1, 20+) |
| Random | 70 | 6 months | 100 | 0 | 193.1 (113.2, 420.3) | 164.3 (104.5, 321.1) | 13.9 (8.1, 20+) |
| Household | 80 | 2 years | 24 | 0 | 183 (138.3, 251.8) | 153.1 (120.0, 202.7) | 13.9 (12.2, 18.6) |
| Random | 80 | 2 years | 15 | 0 | 177 (134.4, 236.4) | 148.0 (114.6, 190.8) | 13.8 (12.2, 20+) |
| Random | 70 | 1 year | 93 | 0 | 162.5 (107.1, 249.3) | 140.4 (99.9, 203.5) | 12.9 (8.7, 20+) |
| Household | 70 | 1 year | 93 | 0 | 159.4 (113.3, 227.8) | 138.8 (101.5, 190.3) | 12.7 (8.7, 18.3) |
| Household | 70 | 2 years | 0 | 0 | 143 (108.1, 191.0) | 121.3 (94.4, 158.0) | 13.2 (1.7, 16.4) |
| Random | 70 | 2 years | 0 | 0 | 138.5 (100.3, 178.6) | 117.3 (88.1, 148.7) | 13.0 (1.6, 17.9) |
| Household | 60 | 6 months | 84 | 0 | 133.8 (80.9, 213.6) | 118.8 (80.1, 179.6) | 12.1 (7.7, 18.4) |
| Household | 60 | 1 year | 41 | 0 | 122.5 (86.8, 165.8) | 108.6 (81.3, 141.2) | 11.4 (8.1, 13.7) |
| Random | 60 | 6 months | 76 | 0 | 120.8 (78.4, 176.3) | 108.8 (75.8, 151.8) | 11.2 (7.0, 18.5) |
| Household | 60 | 2 years | 0 | 0 | 117.3 (78.7, 155.9) | 99.6 (67.8, 128.0) | 12.8 (1.5, 18.8) |
| Random | 60 | 1 year | 23 | 0 | 114.9 (77.6, 152.6) | 102.1 (73.4, 131.5) | 11.2 (7.6, 13.4) |
| Random | 60 | 2 years | 0 | 0 | 110.7 (79.6, 141.1) | 94.1 (71.5, 118.0) | 12.8 (1.5, 20+) |

### **S6.1.2. Results with treatment efficacy of 95% (rather than 90%)**

In MDA strategies with treatment efficacy of 95% (rather than 90%), 80% population coverage, household selection in 5 rounds with 2-year time interval, the probability of achieving a prevalence of less than the stopping threshold increases from 0.64 to 0.89 (**Fig. S*16***). Average DALYs averted per 10,000 people in 20 years increases from 80.5 years to 87 years in MDA strategies with 60% coverage, household-based individual selection in 3 rounds with 6-month time interval when a treatment efficacy of 95% is assumed rather than 90% (**Table S*9***). The probability of scabies elimination increases from 18% to 22% in MDA strategies with 90% coverage, household-based individual selection in 5 rounds with 6-month time interval when a treatment efficacy of 95% is assumed rather than 90% (**Table S*10***).

**Fig. S16:** The proportion of simulation runs with a prevalence of less than or equal to 2% is achieved in differing MDA strategies having a treatment efficacy of 95% (rather than 90%) in scenarios with **an average duration of infestation of 90 days**. The first column (a & c) are the MDA strategies with household-based selection and the second column (b & d) are the MDA strategies with random individual selection. The first row (a & b) are the MDA strategies with three rounds and the second row (c & d) are the MDA strategies with 5 rounds. Each panel is grouped by the population coverage in MDAs and MDA intervals. Each value is calculated from 100 simulation runs.

**Table S9:** Percentage of simulations with <=2% prevalence 1 year after the last MDA round, percentage of simulations with scabies elimination, DALYs averted per 10,000 people (mean, 2.5–97.5 quantiles), and time until prevalence returns to baseline are presented in **MDA strategies with 3 rounds and 95% treatment efficacy** in scenarios with **an average duration of infestation of 90 days.** Time until prevalence returns to baseline is calculated among the runs in which scabies is not eliminated. It is assumed that there is no scabies importation. MDA strategies are ordered from best to worst in terms of (undiscounted) DALYs averted.

| Population selection | Population coverage (%) | MDA Interval | The percentage of runs with <=2% prevalence 1 year after the last MDA round (%) | Percentage of runs with scabies elimination  (%) | DALYs averted per 10,000 people in 20 years  (Mean, 2.5 – 97.5 quantiles) | Discounted DALYs averted per 10,000 people in 20 years  (Mean, 2.5 – 97.5 quantiles) | Time until prevalence returns to baseline  (years) |
| --- | --- | --- | --- | --- | --- | --- | --- |
| Household | 90 | 6 months | 100 | 0 | 254.3 (122.7, 481.9) | 210.5 (110.5, 365.1) | 20+ (8.6, 20+) |
| Random | 90 | 6 months | 100 | 0 | 253.6 (127.3, 459.7) | 209.5 (113.8, 349.4) | 20+ (8.8, 20+) |
| Household | 90 | 1 year | 100 | 0 | 234.7 (120.9, 450.8) | 195.7 (111.6, 339.7) | 18.8 (8.9, 20+) |
| Random | 90 | 1 year | 100 | 0 | 228.3 (123.1, 469.6) | 191.4 (113.6, 354.3) | 18.0 (8.6, 20+) |
| Household | 90 | 2 years | 100 | 0 | 199.4 (131.8, 409.3) | 168.8 (119.0, 307.9) | 13.9 (10.2, 20+) |
| Random | 90 | 2 years | 97 | 0 | 189.7 (121.7, 381.4) | 161.6 (111.6, 290.8) | 13.6 (10.3, 20+) |
| Household | 80 | 6 months | 100 | 0 | 168.5 (90.8, 412.9) | 147.4 (90.6, 316.0) | 13.0 (7.6, 20+) |
| Random | 80 | 1 year | 97 | 0 | 156.3 (90.9, 423.5) | 136.4 (84.5, 319.6) | 12.9 (7.5, 20+) |
| Household | 80 | 1 year | 97 | 0 | 150.9 (95.2, 284.9) | 133.0 (90.5, 231.5) | 12.7 (7.8, 18.8) |
| Random | 80 | 6 months | 100 | 0 | 150.2 (91.1, 226.2) | 133.4 (87.9, 194.7) | 12.5 (6.9, 18.0) |
| Household | 80 | 2 years | 61 | 0 | 135.6 (89.5, 189.5) | 119.7 (84.0, 162.1) | 12.2 (8.4, 17.5) |
| Random | 80 | 2 years | 65 | 0 | 133.8 (94.0, 181.0) | 118.3 (85.1, 156.1) | 12.2 (8.3, 13.7) |
| Household | 70 | 6 months | 90 | 0 | 114.2 (73.5, 183.2) | 104.3 (70.9, 158.6) | 8.8 (6.7, 13.5) |
| Random | 70 | 6 months | 82 | 0 | 110.3 (62.2, 162.4) | 100.5 (62.0, 142.0) | 8.5 (6.2, 13.6) |
| Household | 70 | 1 year | 46 | 0 | 106.2 (70.5, 146.5) | 96.5 (67.2, 130.8) | 8.4 (6.6, 13.6) |
| Household | 70 | 2 years | 3 | 0 | 106 (70.5, 147.7) | 94.5 (66.3, 127.9) | 11.0 (7.5, 13.6) |
| Random | 70 | 1 year | 31 | 0 | 102.8 (62.6, 148.9) | 93.4 (62.7, 132.5) | 8.3 (6.8, 13.4) |
| Random | 70 | 2 years | 4 | 0 | 102 (68.5, 142.2) | 91.4 (64.8, 122.6) | 11.0 (7.6, 13.6) |
| Household | 60 | 6 months | 35 | 0 | 87 (44.5, 120.6) | 80.3 (46.1, 106.8) | 7.7 (6.0, 13.7) |
| Household | 60 | 1 year | 5 | 0 | 85.1 (42.5, 134.0) | 77.9 (46.0, 118.9) | 7.8 (6.2, 13.6) |
| Household | 60 | 2 years | 1 | 0 | 84.6 (55.3, 119.8) | 75.9 (53.2, 102.0) | 8.4 (6.9, 13.5) |
| Random | 60 | 6 months | 17 | 0 | 81.3 (44.7, 117.0) | 75.4 (48.8, 105.7) | 7.4 (5.8, 13.1) |
| Random | 60 | 1 year | 2 | 0 | 80.5 (41.6, 121.4) | 73.6 (43.6, 105.6) | 7.6 (6.1, 13.8) |
| Random | 60 | 2 years | 0 | 0 | 80.5 (39.8, 113.4) | 72.1 (39.8, 98.5) | 8.1 (1.5, 13.2) |

**Table S10:** Percentage of simulations with <=2% prevalence 1 year after the last MDA round, percentage of simulations with scabies elimination, DALYs averted per 10,000 people (mean, 2.5 – 97.5 quantiles), and time until prevalence returns to baseline are presented in **MDA strategies with 5 rounds and 95% treatment efficacy** in scenarios with **an average duration of infestation of 90 days.** Time until prevalence returns to baseline is calculated among the runs in which scabies is not eliminated. It is assumed that there is no scabies importation. MDA strategies are ordered from best to worst in terms of (undiscounted) DALYs averted.

| Population selection | Population coverage (%) | MDA Interval | The percentage of runs with <=2% prevalence 1 year after the last MDA round (%) | Percentage of runs with scabies elimination  (%) | DALYs averted per 10,000 people in 20 years  (Mean, 2.5 – 97.5 quantiles) | Discounted DALYs averted per 10,000 people in 20 years  (Mean, 2.5 – 97.5 quantiles) | Time until prevalence returns to baseline  (years) |
| --- | --- | --- | --- | --- | --- | --- | --- |
| Household | 90 | 2 years | 100 | 6 | 364.5 (235.9, 462.1) | 280.5 (196.7, 345.5) | 20+ (15.9, 20+) |
| Household | 90 | 1 year | 100 | 12 | 355.6 (221.8, 481.5) | 280.0 (192.2, 364.0) | 20+ (13.5, 20+) |
| Household | 90 | 6 months | 100 | 22 | 345.3 (180.1, 486.8) | 274.0 (160.7, 368.7) | 20+ (12.5, 20+) |
| Random | 90 | 1 year | 100 | 16 | 343.3 (196.4, 478.3) | 271.6 (171.2, 360.3) | 20+ (12.6, 20+) |
| Random | 90 | 2 years | 100 | 5 | 342.5 (233.4, 458.1) | 266.4 (191.9, 341.1) | 20+ (14.8, 20+) |
| Random | 90 | 6 months | 100 | 16 | 342.4 (180.6, 487.2) | 271.7 (161.0, 369.2) | 20+ (12.5, 20+) |
| Random | 80 | 6 months | 100 | 0 | 310.2 (152.2, 469.8) | 249.7 (137.0, 356.0) | 20+ (11.1, 20+) |
| Household | 80 | 6 months | 100 | 0 | 306 (162.7, 479.8) | 246.8 (145.7, 361.5) | 20+ (11.4, 20+) |
| Household | 80 | 1 year | 100 | 0 | 300.5 (172.7, 464.8) | 241.1 (151.1, 348.4) | 20+ (12.0, 20+) |
| Random | 80 | 1 year | 100 | 0 | 286 (154.4, 469.0) | 231.2 (135.9, 352.0) | 20+ (11.6, 20+) |
| Household | 70 | 6 months | 100 | 0 | 284 (150.3, 478.7) | 230.7 (135.1, 360.8) | 20+ (10.6, 20+) |
| Random | 70 | 6 months | 100 | 0 | 262.5 (135.8, 462.8) | 215.4 (123.9, 352.3) | 20+ (8.4, 20+) |
| Household | 80 | 2 years | 89 | 0 | 249.8 (174.9, 401.7) | 202.0 (150.2, 298.2) | 18.2 (12.8, 20+) |
| Random | 80 | 2 years | 79 | 0 | 238.6 (173.0, 401.6) | 194.4 (145.8, 300.0) | 17.8 (12.7, 20+) |
| Household | 70 | 1 year | 100 | 0 | 233.2 (135.9, 460.2) | 192.6 (122.8, 344.5) | 18.4 (10.5, 20+) |
| Random | 70 | 1 year | 99 | 0 | 208.4 (123.5, 405.0) | 175.4 (112.1, 304.8) | 16.5 (9.7, 20+) |
| Household | 60 | 6 months | 99 | 0 | 183.5 (98.8, 439.0) | 157.7 (95.3, 332.7) | 13.6 (8.1, 20+) |
| Household | 70 | 2 years | 17 | 0 | 176.5 (133.0, 223.0) | 148.0 (115.3, 180.6) | 13.8 (12.6, 18.7) |
| Random | 70 | 2 years | 16 | 0 | 168.8 (131.2, 226.7) | 142.2 (114.4, 184.8) | 13.6 (11.4, 18.8) |
| Random | 60 | 6 months | 99 | 0 | 163.2 (100.1, 411.6) | 142.1 (93.9, 311.4) | 12.9 (7.9, 20+) |
| Household | 60 | 1 year | 85 | 0 | 159.5 (111.3, 250.5) | 138.1 (103.1, 206.9) | 13.0 (8.6, 18.6) |
| Household | 60 | 2 years | 0 | 0 | 140.2 (110.3, 180.8) | 118.6 (95.8, 148.2) | 13.1 (11.8, 17.7) |
| Random | 60 | 1 year | 72 | 0 | 139.6 (94.9, 197.7) | 122.8 (89.7, 166.5) | 12.3 (8.0, 17.8) |
| Random | 60 | 2 years | 0 | 0 | 131.7 (86.7, 182.5) | 111.7 (78.1, 147.9) | 13.0 (1.5, 17.7) |

### **S6.1.3. Results with individuals not receiving one or two doses of an MDA through lower treatment efficacy**

In this section, we show how people not receiving one or two doses in each round impact efficacy of an MDA strategy and how this can be explained through lower treatment efficacy and population coverage.

Some of the participating individuals in an MDA strategy may prefer to not receive one or two doses of treatment in any given MDA round. This scenario can be considered as a random non-compliance scenario which is different from the systematic non-compliance scenario. The impact of this can be achieved by either decreasing treatment efficacy or population coverage of the given MDA strategy. We present an exemplar scenario (Fig S17.b) where one-third of the selected individuals do not receive both doses (or more than one-third of the selected individuals do not receive one dose) where there is 90% of treatment efficacy of receiving two doses. In this scenario, overall treatment efficacy of 80% of the population in each round decreases from 90% to 60%. Compared to every selected individual receiving both doses (Fig S17.a), decreasing the prevalence to less than the target threshold of 2% is not achieved.


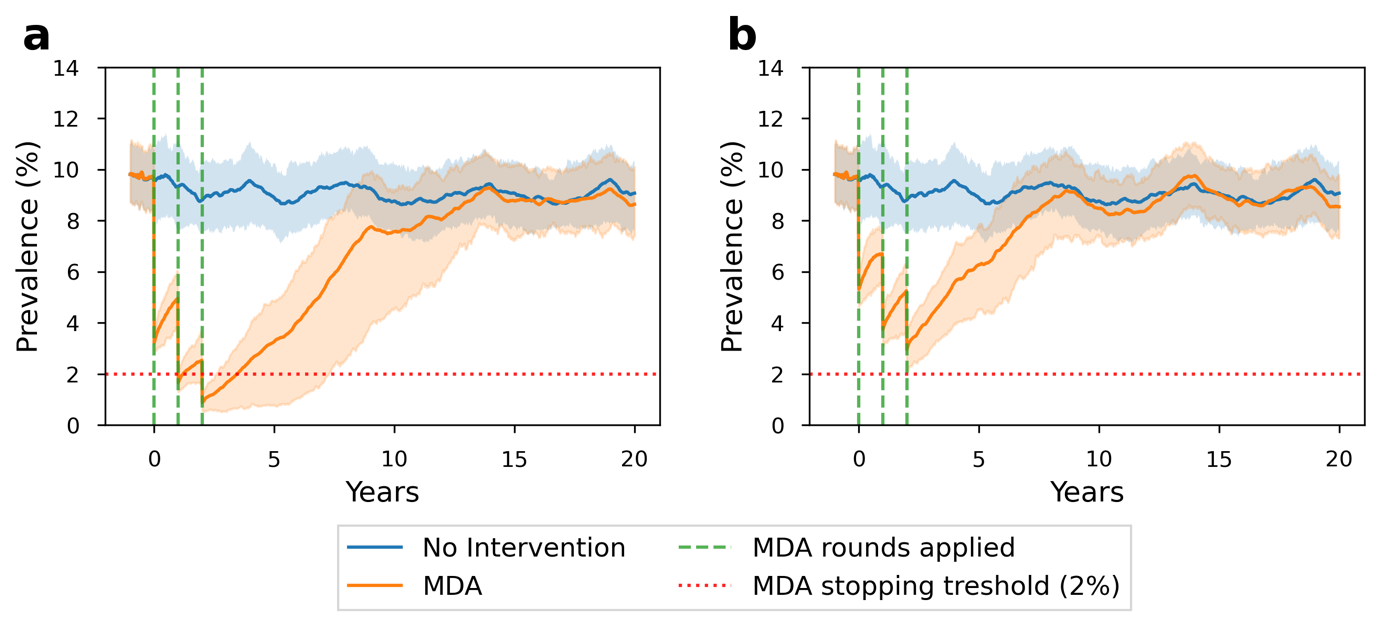


**Fig. S17:** The scabies prevalence in 20 years with MDA strategies consisting of 3 annual rounds, 80% population coverage, random individual selection and (a) 90% treatment efficacy (b) 60% treatment efficacy with an average of duration of infestation of 90 days. The green dashed lines show when MDA rounds are applied. The red dotted lines represent MDA stopping threshold (2%). It is assumed that there is no scabies importation.

Similar results can be achieved through decreasing population coverage rather than treatment efficacy. For example, an MDA strategy with 60% population coverage, 90% treatment efficacy, two doses are received by every individual corresponds to an MDA strategy with 80% population coverage, 90% treatment efficacy, two doses are received by 75% of the participating individuals and none of the doses are received by 25% of the individuals.

## **S6.2. Results with scabies importation**

## **S6.2.1. Results with one scabies case importation each week**

In this section, we present effectiveness of MDA rounds in scenarios with one person infested by scabies outside of the community every week under the assumption of an average infestation duration of 90 days and 150 days. We compare the results with the ‘no importation’ scenarios (**Fig. S*18*** and **Fig. S*19***).

We observe that scenarios with and without scabies importation differentiate the most in terms of scabies prevalence at endemic level. When one weekly scabies case is imported to the population through migration, average scabies prevalence increases from 1% to 3% in scenarios with an average infestation duration of 81 days (**Fig. S*18***).

While it is possible to expect improvements in the healthcare system in the long run without an MDA strategy, it is unlikely to break the normalisation cycle and expect more people to seek treatment without a capacity problem in the healthcare system. For instance, in order to achieve a 20% reduction in the infestation duration without an MDA (difference between red solid and dotted lines in **Fig. S*18***.b), more than 23,000 additional people are needed to seek treatment between year two and three in Monrovia, Liberia.


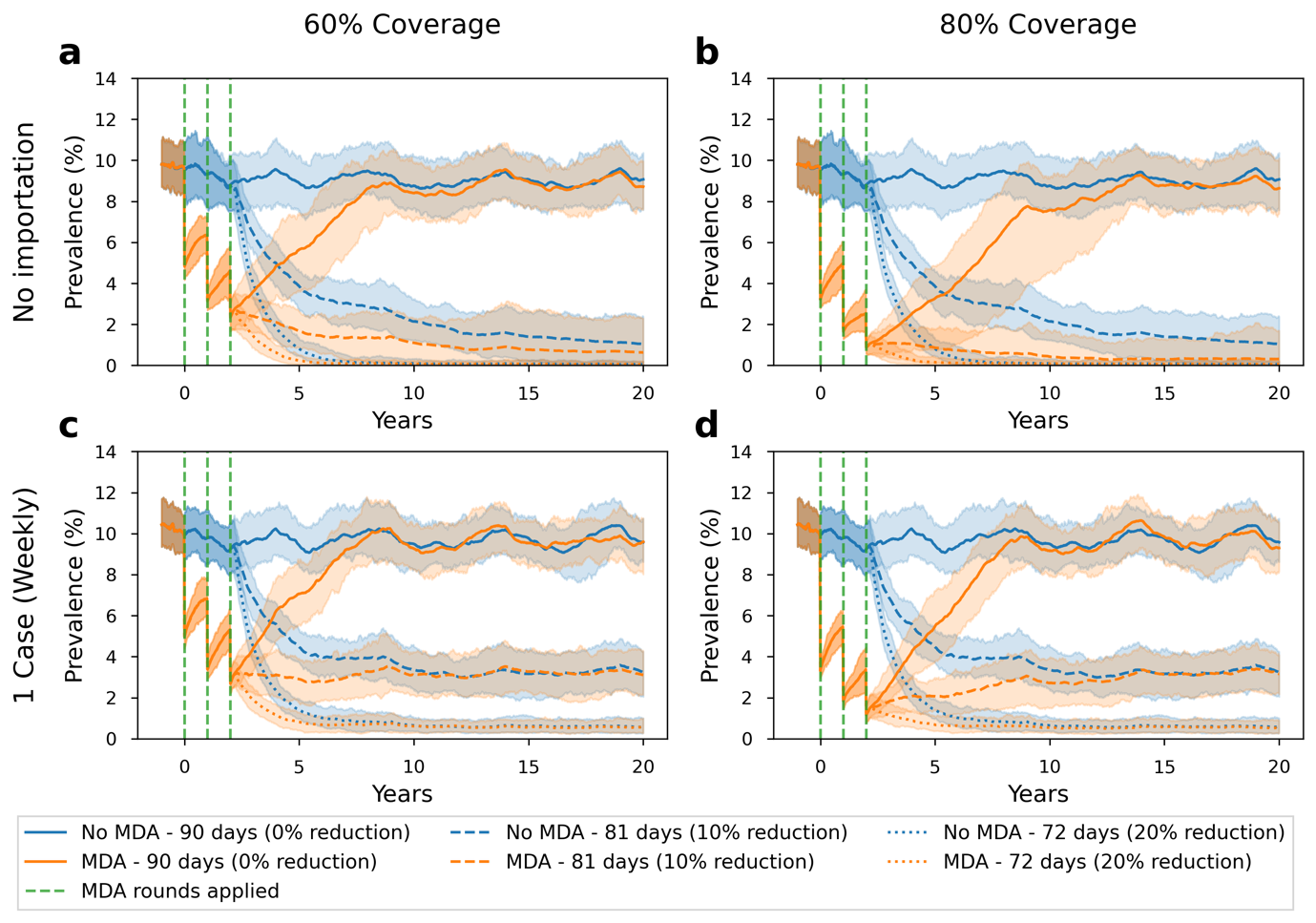


**Fig. S18:** The scabies prevalence in 20 years with (orange) and without (blue) MDA strategy in scenarios (a) 60% population coverage – no importation, (b) 80% population coverage – no importation, (c) 60% population coverage – with scabies importation, and (d) 80% population coverage – with scabies importation **with an average infestation duration of 90 days**. The green lines show when MDA rounds are applied (years 0, 1, and 2). Solid lines show scenarios with an average of 90 days (baseline – 0% reduction) duration of infestation throughout the simulation while dashed and dotted lines show an average of 81 (10% reduction) and 72 (20% reduction) days duration of infestation after year 2, respectively. MDA strategies are applied with random individual selection in 3 annual rounds. In scenarios with scabies importation, there is only one weekly scabies importation. Panel b represents results from Figure 3 in the main manuscript.


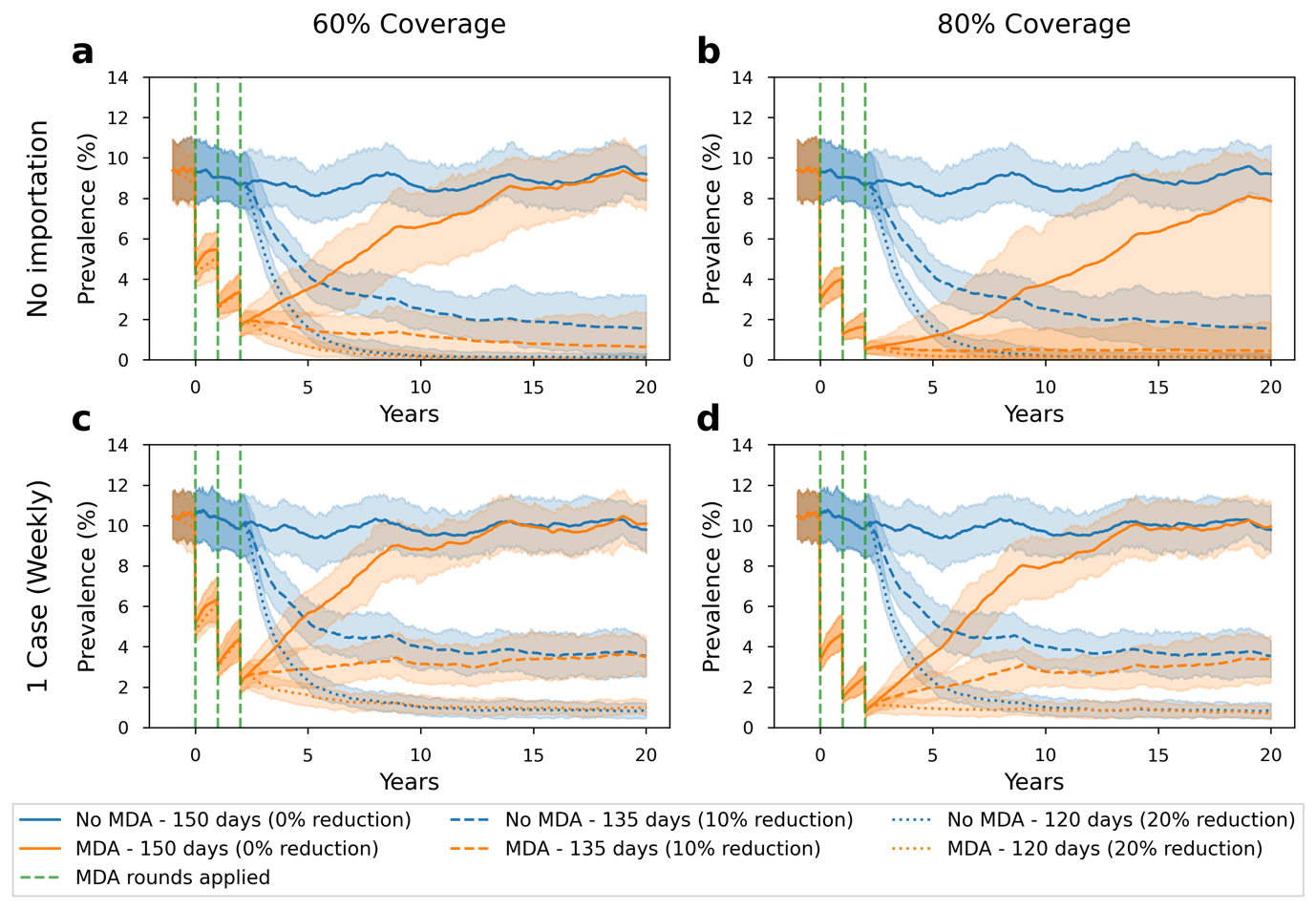


**Fig. S19:** The scabies prevalence in 20 years with (orange) and without (blue) MDA strategy in scenarios (a) 60% population coverage – no importation, (b) 80% population coverage – no importation, (c) 60% population coverage – with scabies importation, and (d) 80% population coverage – with scabies importation **with an average infestation duration of 150 days**. The green lines show when MDA rounds are applied (years 0, 1, and 2). Solid lines show scenarios with an average of 150 days (baseline) of the duration of infestation throughout the simulation while dashed and dotted lines show an average of 135 (10% reduction) and 120 (20% reduction) days of the duration of infestation after year 2, respectively MDA strategies are applied with random individual selection in 3 annual rounds. In scenarios with scabies importation, there is only one weekly scabies importation.

## **S6.2.2. Results with five scabies case importation each week**

In this section, we present effectiveness of MDA rounds in scenarios with five people infested by scabies outside of the community every week under the assumption of an average infestation duration of 90 days. Due to high scabies importation, we recalibrated the transmission parameters to achieve a prevalence around 9.3%.

The results show that when endemic scabies prevalence is maintained by the scabies importation into the community, the long-term effectiveness of MDA decreases as it takes less time for the prevalence to return to the baseline endemic level following MDA (**Fig. S*20***).


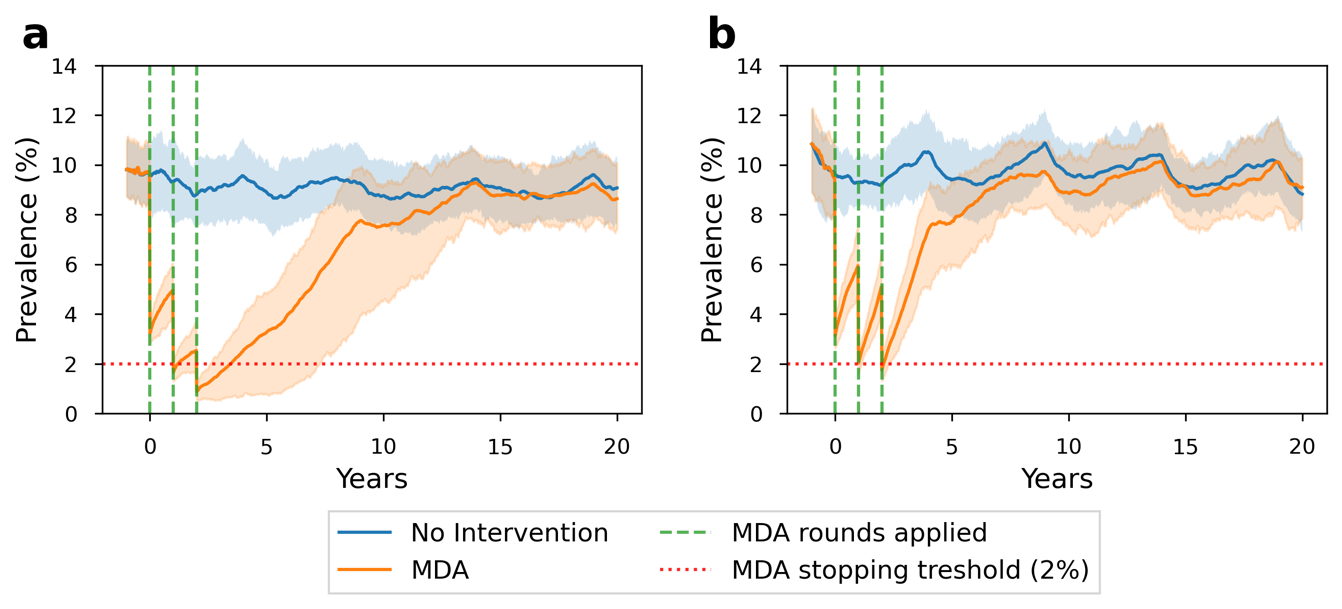


**Fig. S20:** Scabies prevalence over 20 years with (orange) and without (blue) an MDA strategy (a) no scabies importation and (b) a high level of scabies importation (five scabies cases imported each week). Green dashed lines show when MDA rounds are applied (years 0, 1, and 2). MDA strategies were applied with random individual selection in 3 annual rounds and 80% population coverage. Baseline disease transmission parameters in both scenarios have been calibrated to the Monrovia data. Simulation has been run for 10 years in panel a (20 years in panel b) before year 0 to reach an endemic prevalence.

# **S7. Analysis of non-compliance and non-treatment**

## **S7.1 Analysis of systematic non-treatment**

In the manuscript, we provide scenarios under the assumption that on average it takes 90 days for an individual to receive treatment (150 days in Section S5). Here, we focus on systematic non-treatment where some of the individuals systematically do not receive treatment at the base scenario (without MDA). To achieve this, we select 10% of the infected individuals to have an infestation duration of 5 years rather than 90 days (resulting in a population average duration of 9 months of infestation) and recalibrate the disease transmission parameters. Due to the increase in the infestation duration, disease spreads very slowly in the community, therefore, it takes more time to reach an endemic level of prevalence. Therefore, we set the epidemic burn-in period as 60 years rather than 10 years which creates a larger population size and flattens endemic prevalence dynamics (**Fig. S*21***).

The results show that when the endemic disease prevalence is driven by systematic non-treatment, i.e., a small percentage of the infested individuals who remain infested for an average of 5 years (**Fig. S*21***.b).Finding and treating those individuals having scabies for a long time through an MDA strategy can have a more sustained impact because disease transmission is maintained mostly by them transmitting scabies. This finding is also in accordance with the results in Section S5: increasing the duration of infestation decreases the transmission rates, therefore, treating people via an MDA strategy becomes more effective to sustain disease prevalence at lower levels. This finding suggests that whether the endemic prevalence is maintained due to high disease transmission (including household, community transmission and importation of scabies to the community) or high infestation duration determines the long-term effectiveness of MDA.

**
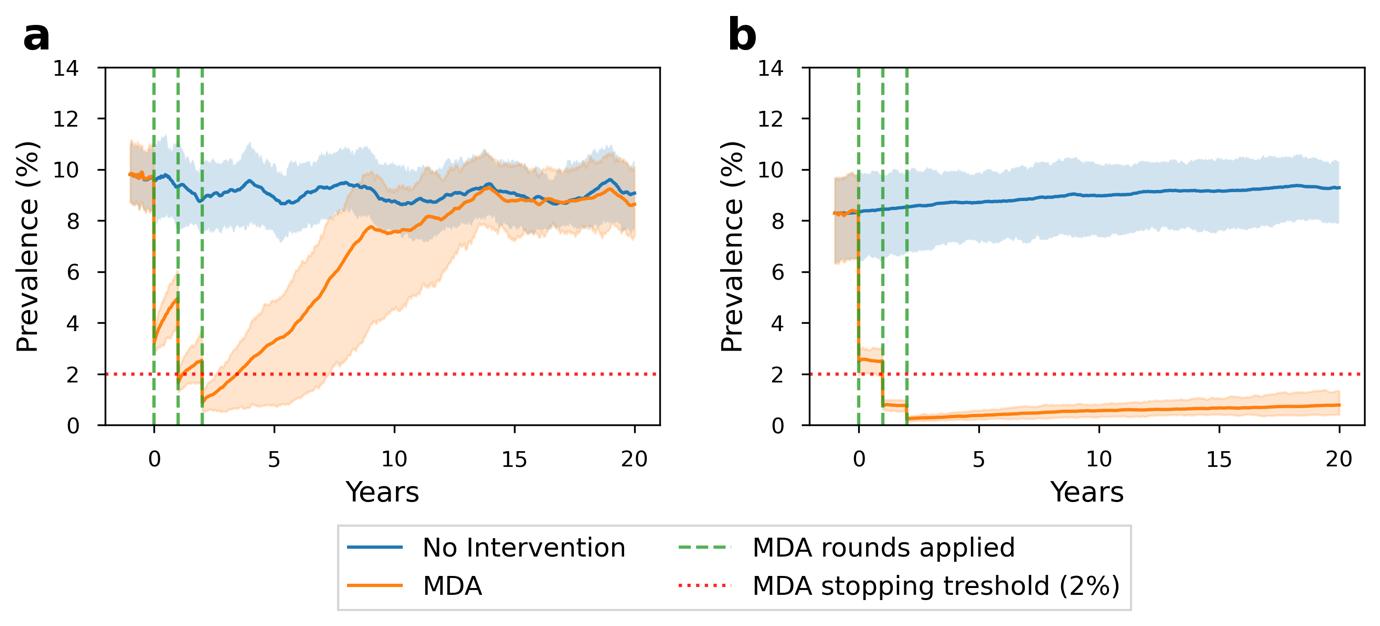
**

**Fig. S21:** The scabies prevalence in 20 years with MDA strategies consisting of 3 rounds, 80% population coverage, random individual selection, and (a) without systematic non-treatment (b) with systematic non-treatment. The green dashed lines show when MDA rounds are applied. The red dotted lines represent MDA stopping threshold (2%). It is assumed that there is no scabies importation.

## **S7.2 Analysis of systematic non-compliance**

We simulated systematic non-compliance by randomly allocation 20% of households to never receive treatment throughout a simulation run. Then, when conducting and MDA, we only treated individuals from households that were not labelled as non-compliant. Given a 0% and 20% systematic non-compliance percentage (0% representing the results in the solid orange line in the main text - Fig 3), for example, systematic non-compliance does not significantly impact the effectiveness of MDA (**Fig. S*22***).

**Fig. S22:** The scabies prevalence in 20 years with MDA strategies consisting of 3 annual rounds, 80% population coverage, and (a) random individual selection without systematic non-compliance (b) random individual selection with systematic non-compliance with an average of duration of infestation of 90 days. The green dashed lines show when MDA rounds are applied. The red dotted lines represent MDA stopping threshold (2%). It is assumed that there is no scabies importation.

Systematic non-compliance with random individual selection results almost corresponds to the household selection results (**Fig. S*22***.b & **Fig. S*23***.b) as by definition, the household selection method selects households and keeps the information of whether a household participates from one round to the following rounds.


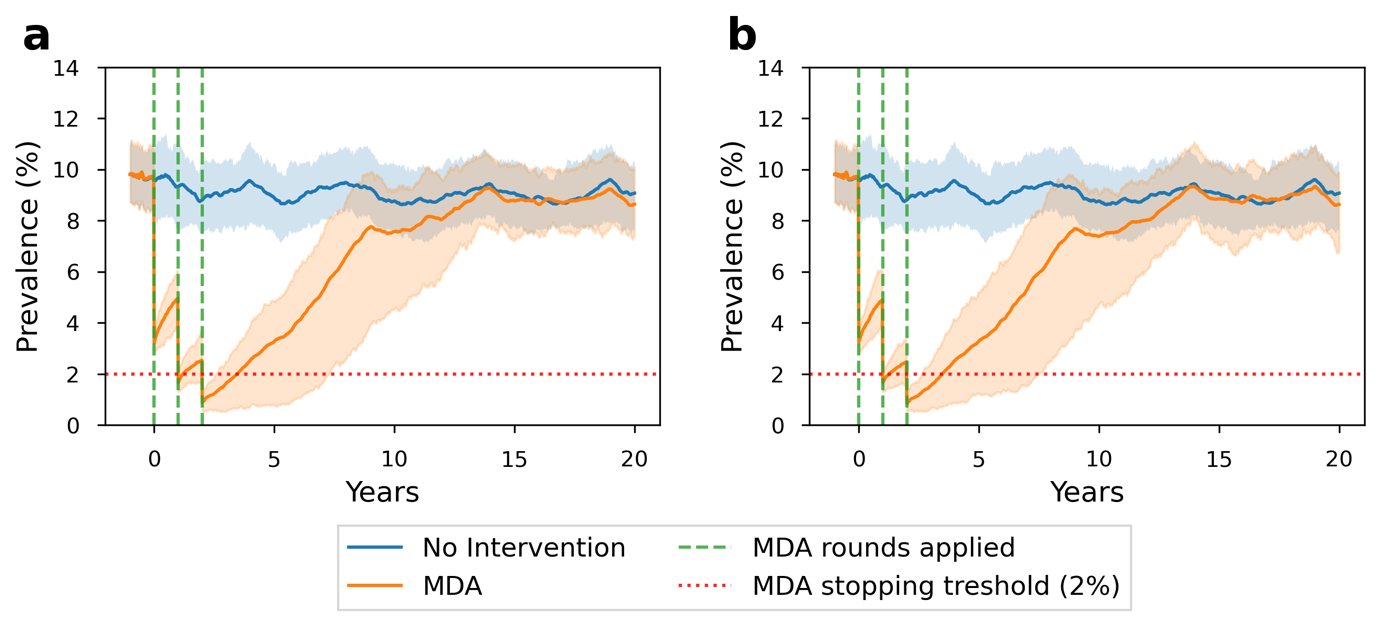


**Fig. S23:** The scabies prevalence in 20 years with MDA strategies consisting of 3 annual rounds, 80% population coverage, and (a) random individual selection (b) household selection with an average of duration of infestation of 90 days. The green dashed lines show when MDA rounds are applied. The red dotted lines represent MDA stopping threshold (2%). It is assumed that there is no scabies importation.

**References**

1. **Geard N, *et al.*** (2015) The effects of demographic change on disease transmission and vaccine impact in a household structured population. *Epidemics*; **13**: 56–64.

2. **Lydeamore MJ, *et al.*** (2019) A biological model of scabies infection dynamics and treatment informs mass drug administration strategies to increase the likelihood of elimination. *Mathematical Biosciences*; **309**: 163–173.

3. **Prem K, Cook AR, Jit M.** (2017) Projecting social contact matrices in 152 countries using contact surveys and demographic data. *PLoS Computational Biology*; **13**: e1005697.

4. **Collinson S, *et al.*** (2020) The prevalence of scabies in Monrovia, Liberia: A population-based survey. *PLoS Neglected Tropical Diseases*; **14**: e0008943.

5. **World Bank Liberia Institute for Statistics and Geo-Information Services** (2017) Household Income and Expenditure Survey 2016 Liberia, 2016 – 2017. Available at: <https://microdata.worldbank.org/index.php/catalog/2986/data-dictionary> (Accessed 27 May 2022).

6. **Lopez AD, *et al.*** (2001) Life Tables for 191 Countries: Data, Methods and Results. 2001.

7. **United Nations Department of Economic and Social Affairs Population Division** (2019) World Population Prospects 2019, Online Edition. Rev. 1. Available at: <http://data.un.org/Data.aspx?d=PopDiv&f=variableID%3A54> (Accessed 27 May 2022).

8. **Gutmann MU, Corander J** (2016) Bayesian optimization for likelihood-free inference of simulator-based statistical models. *Journal of Machine Learning Research*; **17**: 1–47.

9. **Karimkhani C, *et al.*** (2017) The global burden of scabies: a cross-sectional analysis from the Global Burden of Disease Study 2015. *Lancet Infectious Diseases*; **17**: 1247–1254.
